# Neural Correlates of Modal Displacement and Discourse-Updating under (Un)Certainty

**DOI:** 10.1523/ENEURO.0290-20.2020

**Published:** 2021-01-12

**Authors:** Maxime Tulling, Ryan Law, Ailís Cournane, Liina Pylkkänen

**Affiliations:** 1 Department of Linguistics, New York University, New York, NY 10003; 2 New York University Abu Dhabi Institute, New York University Abu Dhabi, Saadiyat Island, Abu Dhabi, United Arab Emirate; 3 Department of Psychology, New York University, New York, NY 10003

**Keywords:** discourse updating, language comprehension, MEG, modal displacement, situation model, theory of mind

## Abstract

A hallmark of human thought is the ability to think about not just the actual world but also about alternative ways the world could be. One way to study this contrast is through language. Language has grammatical devices for expressing possibilities and necessities, such as the words *might* or *must*. With these devices, called “modal expressions,” we can study the actual versus possible contrast in a highly controlled way. While factual utterances such as “There is a monster under my bed” update the here-and-now of a discourse model, a modal version of this sentence, “There might be a monster under my bed,” displaces from the here-and-now and merely postulates a possibility. We used magnetoencephalography (MEG) to test whether the processes of discourse updating and modal displacement dissociate in the brain. Factual and modal utterances were embedded in short narratives, and across two experiments, factual expressions increased the measured activity over modal expressions. However, the localization of the increase appeared to depend on perspective: signal localizing in right temporoparietal areas increased when updating the representation of someone else’s beliefs, while frontal medial areas seem sensitive to updating one’s own beliefs. The presence of modal displacement did not elevate MEG signal strength in any of our analyses. In sum, this study identifies potential neural signatures of the process by which facts get added to our mental representation of the world.

## Significance Statement

When we say things like “There might be a monster under my bed” we distance ourselves from the observable here-and-now and imagine how the world could be. Normally, we are easily able to distinguish reality from mere possibility, but we know very little about the neural mechanisms that allow us to do so. Our research shows that the brain responds differently to utterances about the here-and-now compared with utterances conveying possibilities. This means that our brains rapidly separate factual information from hypothetical information, raising interesting new questions about the representation of possibilities in discourse comprehension. By identifying the neural correlates of updating discourse representations, we pave the way for future research on the processing and representation of non-factual discourse.

## Introduction

Speculating about possibilities employs our unique human capacity to displace from the here-and-now (Hockett, 1959; Bickerton, 2008; Suddendorf et al., 2009). We can express possibility using “modal expressions” like “There *might* be a monster”, shifting our perspective from the immediate present to a hypothetical scenario. Other cognitive abilities that shift into alternative perspectives, like thinking about the past or future and conceiving the viewpoints of others, seem to share a brain network consisting of hippocampal and parietal lobe regions (Buckner and Carroll, 2007; Mullally and Maguire Sinéad, 2014). However, we know surprisingly little about the neural mechanisms involved in modal displacement. While factual statements like “There is a monster” update our beliefs about a situation, modal utterances indicate uncertainty instead. Are the mental operations of discourse updating and modal displacement dissociable in the brain? Here, we investigated the neural correlates of integrating factual and modal utterances into an existing discourse representation.

## Cognitive Processes Involved with Comprehending Discourse

When comprehending discourse, we represent the perspective, place, and time of the discussed situation (van Dijk and Kintsch, 1983; Zwaan and Radvansky, 1998) and distinguish between facts and possibilities compatible with the here-and-now of this alternative reality. Consider this scene from Ovid’s tale about the ill-fated lovers Pyramus and Thisbe:

When a lioness, bloody from hunting, approaches, Thisbe flees into a cave, losing her shawl in the process. As Pyramus encounters the lioness hovering over Thisbe’s bloodstained shawl with his lover nowhere in sight, he quickly concludes she **must** have been devoured by the beast.

All but the underlined sentence are factual claims made about the actual state of affairs (Stalnaker, 1996). We use these utterances to build a mental situation model, which is dynamically updated as new information becomes available (Glenberg et al., 1987; Morrow et al., 1989; Zwaan and Madden, 2004). Maintaining these discourse models elicits activation in the medial prefrontal cortex (mPFC), posterior cingulate cortex (PCC), and temporoparietal areas (Xu et al., 2005; Speer et al., 2007; Yarkoni et al., 2008; Whitney et al., 2009). To interpret the narrative above, we also engage in higher order cognitive processes such as modal displacement and Theory of Mind (ToM) reasoning (Premack and Woodruff, 1978). ToM is the ability to represent someone else’s belief state separately from our own, allowing us to understand how Pyramus induced that Thisbe died, although we know she is still alive. Pyramus based his conclusion on indirect evidence (the bloody shawl), signaling with the modal verb *must* that the devouring is not actual or known. Modals like *must* or *may* allow reasoning about open possibilities compatible with a situation (Kratzer, 1981, 2012; von Fintel, 2006; Phillips and Knobe, 2018).

**Figure 1.**
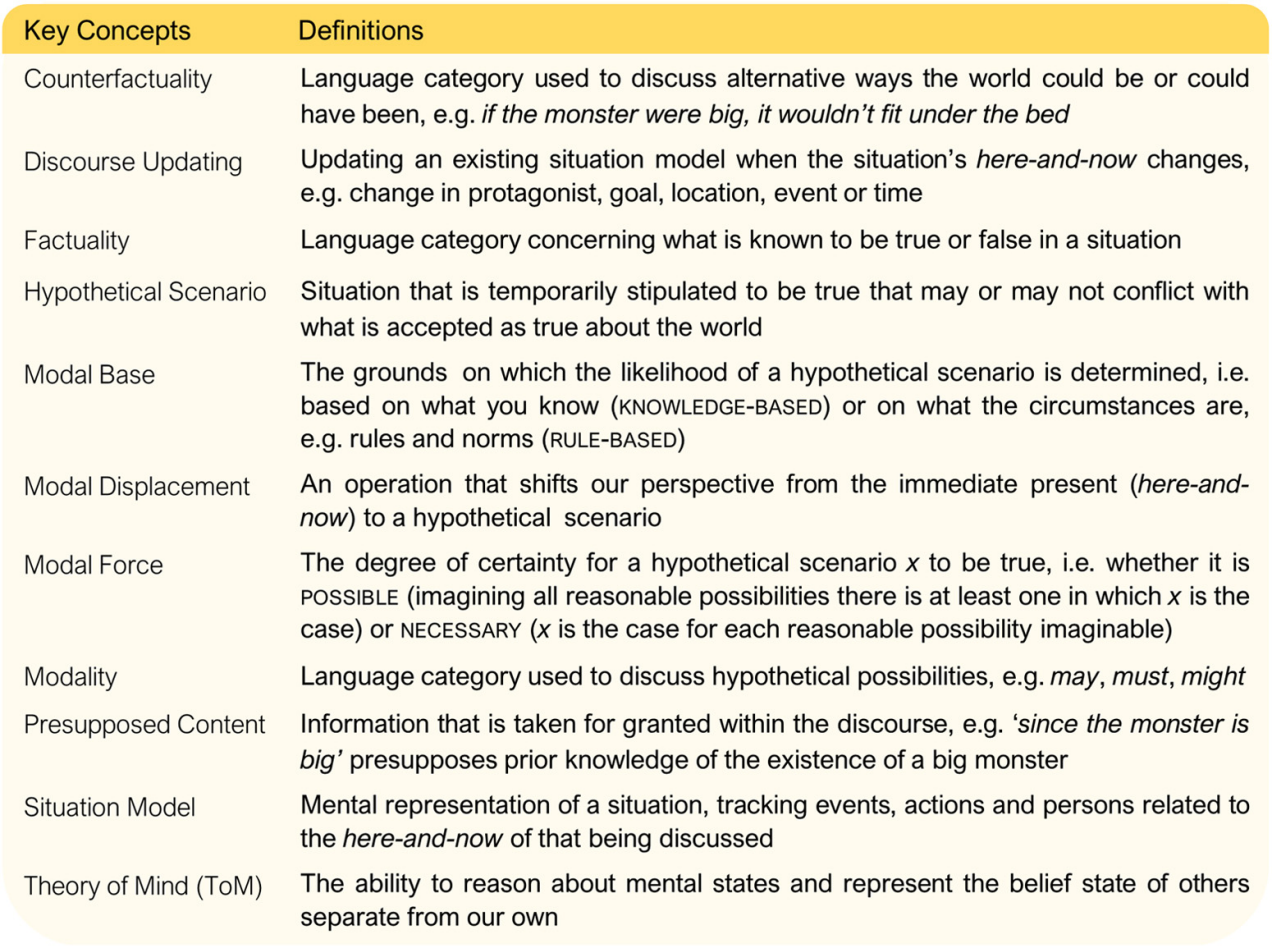
Table containing key concepts and definitions as used in this paper.

Since ToM and modal displacement both require a representation that is different from the actual situation (Phillips and Norby, 2019), they may recruit overlapping brain areas. While there has been no systematic study of the neural bases of modal processing, ToM tasks are consistently reported to activate the dorsal/posterior inferior parietal lobule (IPL), temporoparietal junction (TPJ), mPFC, PCC, and rostral anterior cingulate cortex (rACC; Mahy et al., 2014; Schurz and Perner, 2015; Koster-Hale et al., 2017). In particular, the right TPJ (rTPJ) seems involved in representing other’s mental state (Saxe and Wexler, 2005; Saxe and Powell, 2006; Vistoli et al., 2011), although some suggest this activity may be attributable to more domain general cognitive processes such as reorienting attention (Decety and Lamm, 2007; Corbetta et al., 2008; Mitchell, 2008; Rothmayr et al., 2011). Definitions of the key concepts used throughout this paper are provided in Figure 1.

## This Study

**Figure 2.**
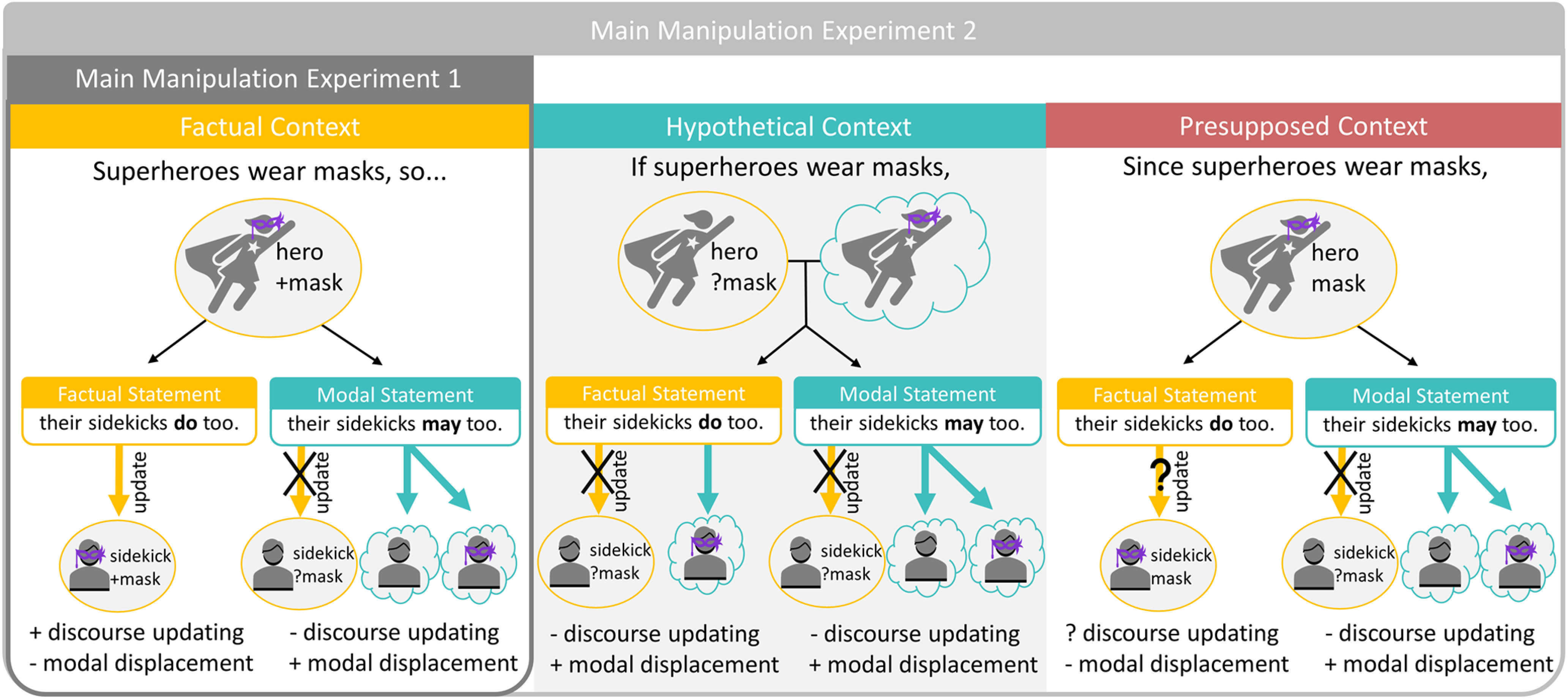
Simplified illustration of main manipulations of experiments 1 and 2. Model of operations assumed to be present during the processing of factual (yellow) and modal (teal) statements (simplified from actual stimuli). Experiment 1 contrasts factual and modal statements in a factual discourse context, while experiment 2 varies whether the discourse context is factual, hypothetical, or presupposed. Updating of the discourse situation model (round) is expected to take place under certainty (in factual contexts with a factual update). Both modal (*may*) and conditional expressions (*if superheroes wear masks*) evoke hypothetical situations (cloud) involving modal displacement. Since the presupposed context marks information already known, we are not sure whether updating would take place.

How do our brains distinguish between information that states facts versus information that only conveys possibilities? We investigated the differences between factual and modal language comprehension in two experiments (Fig. 2). We used magnetoencephalography (MEG), providing us with high temporal resolution and relatively good spatial localization of brain activity during sentence comprehension. Experiment 1 investigated the neural bases of discourse updating and modal displacement by contrasting sentences that contain modal verbs against sentences containing the factual verb ‘do’ embedded in short narratives. In experiment 2, we further investigated under which conditions discourse updating takes place by manipulating the certainty of the sentential context in which the target verbs (factual vs modal) were embedded: factual (certain), conditional (uncertain), or presupposed (already known). Discourse updating should take place under actual situational changes (e.g., when new factual information is added to a factual context), but not when novel information is hypothetical (modal conditions) or when the entire context is hypothetical (conditional context). Modal displacement should occur whenever utterances postulate hypothetical possibilities.

## Materials and Methods

### Experiment 1

#### Participants

A total of 26 right-handed, native English speakers participated in the experiment (four males) taking place at the New York University’s New York (NY) campus. One participant was excluded from further analysis for having an accuracy lower than 70% on the behavioral task. The age range of the remaining 25 participants was 19–52 years old (M = 25.7, SD = 7.46). All participants had normal or corrected to normal vision, no history of neurologic impairment and provided informed written consent.

#### Stimuli

We developed an experimental paradigm where we contrasted the modal verbs *may* and *must* against the factual auxiliary verb *do*. In order to have *do* naturally appear in the same position as *may* and *must*, our sentences contained verb phrase (VP) ellipsis, e.g., “Normally only knights sit at the round table, but the king says that the squires *may*/*must*/*do* <sit at the round table> too.” While the verb *do* indicates factuality, modals indicate hypothetical scenarios that are compatible with the actual world given someone’s knowledge or the set of circumstances. We specifically chose to use the modal expressions *may* and *must* because they vary among two dimensions: modal force and modal base. Modal force refers to the likelihood of a hypothetical situation, i.e., whether it is deemed a possibility (*may*) or a necessity (*must*). The modal base denotes what we base this likelihood assessment on: our knowledge or the circumstances, e.g., rules/norms. The modals *may* and *must* are ambiguous in allowing for both a knowledge-based (e.g., “Given what I know, there may/must be a monster under my bed”) and a rule-based reading (e.g., “Given what the rules are, you may/must eat your dinner now”). Using such ambiguous modals, we could compare the effect of modal base without varying the form of the target item.

**Figure 3.**
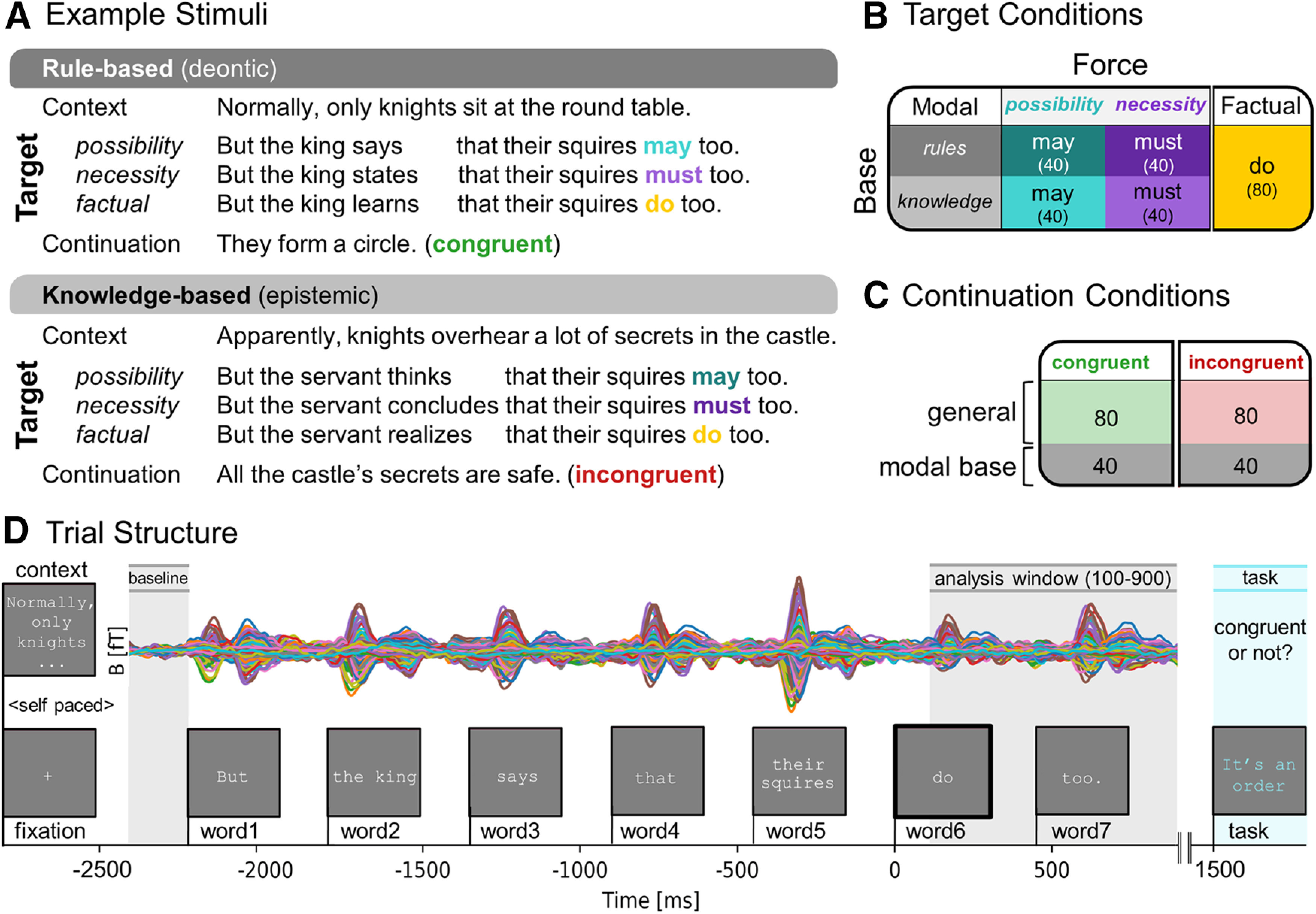
Design and procedure experiment 1. ***A***, Example stimuli set. Short narratives consisted of three parts. A context sentence biasing toward a rule-based or knowledge-based modal interpretation was followed by the target sentence containing one of the target verbs varying in force (possibility, necessity, or factual). The third continuation sentence was either congruent or incongruent with prior sentences. Details on controlled between-stimuli variation can be found in Extended Data Figure 3-1. ***B***, Experimental design with number of items per condition in brackets (total = 240). The stimuli vary along two dimensions: Modal Base (rules, knowledge) and Force (possibility, necessity, factual). ***C***, Continuation conditions. Half of the continuations are incongruent with the previous sentences. One third tap into modality and are congruent or incongruent with the modal base of the previous sentences. ***D***, Trial structure with evoked MEG responses in femtotesla (fT) from one participant. A context sentence was displayed until participants pressed a button. After a fixation cross (300 ms), the target sentence was displayed word-by-word for 300 ms each followed by a 150-ms blank screen. The continuation sentence was displayed with a 600-ms delay, and participants indicated by button press whether this was congruent or incongruent with the prior story. Time windows for baseline correction (−2450 to −2250 ms) and statistiacal analysis (100−900 ms) are relative to the target verb (word6) onset.

We constructed 40 sets of short English narratives. Each story consisted of three sentences, starting with a context sentence designed to either bias toward a knowledge-based (epistemic) scenario, or a rule-based (deontic) scenario. The context sentence was followed by a target sentence and each story ended with a final task sentence that was either congruent or incongruent with the previous two sentences (Fig. 3*A*). The target sentences contained the target modal verb (the possibility verb *may* or the necessity verb *must*) and were compared against the factual condition containing the verb *do*. In the context sentence a property or habit was introduced that applied to one group (e.g., “knights sit at the round table”), and the target sentence indicated this was also (possibly) the case for another group (e.g., “their squires do/may/must too”). Each stimulus set therefore consisted of six sentences (2 × 3, Base: [knowledge, rules] × Force: [possibility, necessity, factual]) adding up to a total of 240 sentences for all 40 stimuli sets (Fig. 3*B*). The third sentence of the story was a task sentence either congruent (50%) or incongruent (50%) with the prior two sentences. One third of the task sentences were specifically tapping into the congruency of the modal base (Fig. 3*C*). Across conditions, how often task items were congruent or incongruent with the preceding sentences was controlled for, as was how often questions tapped into information obtained from the context or target sentence.

10.1523/ENEURO.0290-20.2020.f3-1 Extended Data Figure 3-1 Details on controlled between-stimuli variation experiment 1. The target sentences were identical in structure, e.g., “But the king says that their squires may too” but varied in controlled manner in the following five ways. ***A***, Overview of the variation in count of used connectives (*and*, *but*, and *so*) across modal bases. ***B***, Variation of nouns (main subject) across modal base conditions in average length (in letters), average lexical frequency, average log lexical frequency, number of syllables, and number of morphemes. ***C***, Variation of the determiners used to refer to the embedded subject: the, a long-distance pronoun (LD) referring to a referent in the prior context sentence or a short-distance pronoun (SD) referring to a referent in the target sentence. ***D***, Variation of the elided VP across modal base conditions in average length (in words and letters), percentage of VPs that included verbs indicating a state (in contrast to an event), percentage of verbs taking two arguments (transitive) versus verbs that take one argument (intransitive), average syntactic node count [how many phrase nodes are present counting phrases containing a noun (NP), verb (VP), adjective (AP), preposition (PP), and infinitive (IP)] and average syntactic complexity (maximum amount of nodes opened at the same time), e.g., *to see dusty books at the library* includes five syntactic phrases [IP to [VP see [AP dusty [NP books]]]] [PP at the library] and has at most four nodes open at the same time. ***E1***, List of different embedding verbs used with count of usage across modal bases. ***E2***, Variation of embedding verbs used across modal base conditions in average length (in letters), average lexical frequency, average log lexical frequency, number of syllables, and number of morphemes. Download Figure 3-1, DOC file.

All target sentences had the same sentence structure: connective (but/and/so)| the | noun.sg | verb1 | that | determiner | noun. pl | target (may/must/do) | <elided VP> too. The embedded clause of the sentence (introduced by *that*) was kept consistent across all conditions. We controlled for between-item variation in the other parts of the stimuli along the following dimensions: the count of different connectives and determiners among the modal base conditions, the average length, frequency, number of syllables and morphemes of noun.sg among different modal base conditions, and the average length (in words and letters), stativity, transitivity and structural complexity of the <elided VP> material in the target sentence across different base conditions (see Extended Data Fig. 3-1). The information on lexical frequency and morpheme length was obtained from the English Lexicon Project (Balota et al., 2007). Within the modal base dimension, the target sentences only varied in the embedding verb (verb1) to support biasing the reading of the target modal verb. Embedding verbs were divided into three categories occurring with knowledge-based, rule-based or factual targets. Each verb category contained 12 different verbs, which were repeated maximally seven times across the entire experiment. Between the two base conditions, the knowledge-based and rule-based sentences also differed in their preceding context sentence and subject, to help bias the interpretation of the ambiguous modals *may* and *must*. In order to encourage the rule-based reading, the context introduced an event that was compatible with both permission or obligation (e.g., sitting at the royal table), and the target sentence introduced a third person subject that was in an authority position over the sentence object (e.g., a king over squires). In order to encourage the knowledge-based reading, the context introduced an event that was very unlikely to be permitted or obliged (e.g., overhearing secrets) and the target sentence introduced a subject that was in a bystander position to the event (e.g., a servant). By embedding the target utterance into the perspective of a third person subject, the assessment of the modal force (whether something was possibly, necessarily or factually true) was linked to the perspective of this character.

The effectiveness of the biasing conditions was tested with a survey on Amazon Mechanical Turk made with the help of Turktools (Erlewine and Kotek, 2016). For this norming, the target sentences containing modal verbs (160 items in total) were adjusted so that unambiguous adjectives replaced the ambiguous target modal verbs. Knowledge-based *may* was replaced with *are likely to*, knowledge-based *must* with *are certain to*, rule-based *may* with *are allowed* to and rule-based *must* with *are obliged* to. e.g., the target sentence “But the king says that the squires may too” became “But the king says that the squires are allowed to as well.” These unambiguous target sentences were then displayed with their preceding context sentence and a gap substituting the adjective. Participants (*n* = 320) were asked to choose which of four options (*obliged*, *allowed*, *likely*, and *certain*) would fit the gap best. Each target sentence was judged 32 times across all participants. The experiment took ∼2–4 min, and participants were paid $0.20 for completing the experiment. Each participant completed 25 sentences, comprised of 20 test items and five filler items that served as an attention control, in random order counterbalancing for condition. Results were excluded from participants that indicated to not have English as a native language (*n* = 17) and from participants that made >1 mistake on the filler items (*n* = 6). For the responses of the remaining 297 participants, we noted whether the modal base of their response (*allowed* and *obliged =* rule-based, *likely* and *certain* = knowledge-based) matched the intended modal base of the target items or not. For each item, we calculated the average percentage of matches with the intended modal base (bias score), and only approved an item for the experiment if its bias score was 70% or higher. This norming happened in two parts. In the first round, all 160 items were tested, and 137 items were accepted. The remaining 23 items had a bias score below the 70% threshold and were altered to improve their bias. In the second round, these 23 items were re-tested (now mixed with a random selection of the previously approved items) and judged with the same criteria. This time 18 items were accepted, and five scored below the 70% threshold. The five items that did not pass the norming experiment were altered again with the help and approval of several native speakers, and then included into the experiment.

The lexical frequency of knowledge-based (epistemic) and rule-based (deontic) readings of *may* and *must* are not evenly distributed in written American English: the verb *may* is knowledge-based ∼83% of the time (Collins, 2007), while *must* is knowledge-based 16% of the time (Hacquard and Wellwood, 2012), in all other cases the verb has a circumstantial base that includes rule-based meanings. While these lexical frequency differences may have an effect on the processing of the individual items, we expect that grouping the different levels of the force (grouping *knowledge-based* and *rule-based* responses together) or modal base manipulation (grouping *possibility* and *necessity* responses together) should wash out any effects of this imbalance.

#### Procedure

Before recording, the head shape of each participant was digitized using a FastSCAN laser scanner (Polhemus). Additionally, we recorded the location of three fiducial locations (the nasion, and left and right preauricular points) and five reference points for purposes of co-registration. Before participants entered the MEG-room they received verbal instructions and did a short practice block (of eight trials). Data collection took place in a magnetically shielded room using a whole-head MEG system (157 axial gradiometer sensors, three reference magnetometers; Kanazawa Institute of Technology, Nonoichi, Japan). Before the experiment, we taped five marker coils on the location of the digitized reference points that help establish the position of the subject’s head before and after the experiment. During the experiment, the participant comfortably lay down in the MEG machine, reading from a screen located ∼50 cm away with dimmed lights. Text was displayed in a fixed-width Courier New font on a light gray background.

In the experiment, participants were asked to silently read and comprehend short stories consisting of three sentences presented with PsychoPy (Peirce, 2009). The first sentence (context) was displayed as a whole. Participants read this sentence at their own pace and pressed a button to continue. Then a fixation cross (300 ms) followed and after a 300-ms blank screen the target sentence was presented using Rapid Serial Visual Presentation. Participants were presented with English sentences of nine words, mostly one word at the time, with the exception of determiner-noun pairs, which were presented together so that the sentence was divided into seven parts (called “words” from now on). The display time for all words was 300 ms. Every word was preceded by a blank screen of 150 ms. This was followed by a short third sentence in blue that was either congruent with the previous sentence or incongruent (50%). The continuations were designed such that they targeted the comprehension of different parts of the story (encouraging participants to read the entire narrative with care). One third of the continuations tapped into the modality of the target sentence, in which the continuation is congruent with the modal base (e.g., a sentence about obligation followed by “their mother told them to”) or incongruent with the modal base (e.g., a sentence about obligation followed by “she’s probably right”). We included this manipulation to be sure that participants are paying attention to the fine meaning of the modal target verb. The participant’s task was to press one button with their middle finger for continuations that “made sense” and another button with their index finger if the continuations “did not make sense,” after which the next trial started. The participants were instructed to move and blink as little as possible during the task. The trial structure is displayed graphically in Figure 3*D*.

The experiment consisted of 240 trials in total. The trials were divided into six separate blocks (containing one item per stimuli set) by a balanced Latin square design and randomized within blocks. Each block consisted of 40 sentences and was presented into two parts during the experiment, resulting into 12 blocks which took ∼3–7 min each. In between blocks, participants were informed about their overall accuracy. Participants were free to rest in between blocks and were paid $15 (NY) per hour.

#### Data acquisition

MEG data were sampled at 1000 Hz with an online 200-Hz low-pass filter. The signal was offline noise reduced in the software MEG160 (Yokogawa Electric Corporation and Eagle Technology Corporation) using the signal from the three orthogonally-oriented reference magnetometers (located within the machine, but away from the brain) and the Continuously Adjusted Least-Squares Method (Adachi et al., 2001). Further preprocessing and analysis was performed making use of MNE-Python (Gramfort et al., 2013, 2014) and Eelbrain (Brodbeck, 2017). First, MEG channels that were unresponsive or clearly malfunctioning (separating from all other channels) during the session were interpolated using surrounding channels (6% of the channels in total underwent interpolation, 7–19 channels per participant). We extracted epochs from −2450 to 900 ms relative to the onset of the target verb, which included the entire sentence. The epochs were corrected for the delay between presentation software timing and stimulus presentation, by taking into account the average delay as measured with a photodiode. The data were filtered offline with a bandpass filter between 1 and 40 Hz. Eye blinks and heartbeat artefacts were removed by the use of independent component analysis (ICA) via the “fastICA” option implemented in MNE Python (Gramfort et al., 2014). Additionally, we removed a known artifact pattern (“the iron cross”) that was present at that time across all NY recordings because of an electromagnetic noise source from nearby cables. Any epoch that had a sensor value that was higher than 3 pT or lower than –3 pT was automatically rejected. Additionally, trials were rejected after visual inspection if multiple channels were affected by obvious noise patterns that exceeded the boundaries of the epoch’s window. In total, this resulted in a trial-rejection rate of 4.6% across the experiment. Baseline correction was performed using data from the 200 ms before the first word of the sentence.

The location of sources was estimated by co-registration of the digitized head shape with the FreeSurfer average brain (Fischl, 2012). A source space containing 2562 sources per hemisphere was constructed for each subject, and a forward solution was created with the Boundary Element Model method. The inverse operator was calculated based on the covariance matrix from the 200-ms prestimulus baseline period of the cleaned trials. This inverse operator was applied to the average evoked responses to obtain a time course of minimum norm estimates at each source for each condition signal-to-noise-ratio (SNR = 3). The direction of the current estimates was freely oriented with respect to the cortical surface, and thus all magnitudes were non-negative. The source estimates were then noise-normalized at each source (Dale et al., 2000), generating dynamic statistical parameter maps (dSPM) that were used in statistical analyses.

#### Statistical analyses

##### Behavioral data

Responses and reaction times to the 6000 (25 × 240) congruency decisions were collected and overall accuracy was determined based on the responses to all items. The overall accuracy was used to exclude participants if they scored below 70%. We also examined the accuracy of the 2000 modal task items.

##### MEG data

MEG data were analyzed both with a region of interest (ROI) analysis and with a full-brain analysis, given the explorative nature of our question.

##### ROI analysis

Since there is no prior neuroimaging work on the processing of modals, our ROIs were defined based on previous literature looking at the neural bases of ToM (Mahy et al., 2014; Schurz and Perner, 2015; Koster-Hale et al., 2017), and included the inferior parietal sulcus (IPS), TPJ (bilaterally), superior temporal sulcus (STS), PCC, rACC, and mPFC bilaterally. These functional regions were translated into labels for (bilateral) areas mapped onto the FreeSurfer aparc (Desikan et al., 2006) parcellation (Table 1). Each source current estimate was mapped onto a parcellation, and then averaged over all the sources in each ROI.

**Table 1:** Overview of ROIs based on the aparc parcellation, with approximately corresponding Brodmann areas (BA) and number of sources

Label	Aparc	BA	Number of sources
Inferior parietal sulcus (IPS)	Superiorparietal	7	162
Temporoparietal junction (TPJ)	Supramarginal + inferiorparietal	39 + 40	278
Superior temporal sulcus (STS)	Superiortemporal	22	108
Posterior cingulate cortex (PCC)	Posteriorcingulate	23 + 31	49
Rostral anterior cingulate cortex (rACC)	Rostralanterior-cingulate	24 + 32	15
Ventromedial prefrontal cortex (vMPFC)	Medialorbitofrontal	25 + 10 + 11	44

The effect of the experimental manipulations on our ROIs was assessed with a cluster-based permutation test (Maris and Oostenveld, 2007), aimed to identify temporal clusters that were affected by our experimental paradigm, corrected for multiple comparisons. We performed a temporal cluster-based permutation mass univariate 2 × 3 repeated-measures ANOVA with factors Modal Base and force. Since we had no clear predictions about the possible timing of an effect, we used the generous time window of 100–900 ms after the target verb’s onset. Since several trials got rejected during data preprocessing, to ensure comparable SNR across conditions we equalized trial count across conditions (M = 36 trials/condition, range = 31–39 trials/condition)

Our temporal permutation clustering test was performed in Eelbrain 0.27.5 (Brodbeck, 2017) with a standard procedure. An uncorrected ANOVA was fitted at each time point in the analysis time window (100–900 ms). Temporal clusters were formed and chosen for further analysis when *F* statistics corresponded to significance exceeded the critical α-level of 0.05 (uncorrected) for contiguous time points of at least 25 ms. A test statistic corresponding to the cluster magnitude was then determined by summing over all the *F* values contained within them and selecting the largest of the cluster-level statistics. Conditions were re-labeled, and test statistics were calculated for each subject for 10,000 times to form a null distribution of the test statistics. The observed clusters were compared with this null distribution and were assigned corrected *p* values reflecting the proportion of which random partitions resulted in an *F* statistic greater than the observed *F* statistic. Since in this method, the time point clusters initially chosen for further analysis are uncorrected, the borders of the clusters should be interpreted as having an approximate nature, not making claims about the *exact* latency or duration of any effects (see Sassenhagen and Draschkow, 2019). Finally, to also correct for comparisons across multiple ROIs, we applied a false discovery rate correction for multiple comparisons (Benjamini and Hochberg, 1995).

##### Whole-brain analysis

To complement our ROI analysis, we conducted a full brain analysis, which both described the full spatial extent of any effects observed in the ROI analysis and provided us with information about any effects not captured by the ROI analysis. We performed a spatiotemporal clustering test almost identical to the temporal cluster test described above, only now without averaging sources within an ROI. Instead, an *F* statistic was calculated for each time point in each source, and spatiotemporal clusters were identified where significance exceeded a *p* value of 0.05 for at least 10 spatially contiguous sources and for at least 25 ms. Again, following Sassenhagen and Draschkow (2019), the temporal and spatial properties of the identified significant spatiotemporal clusters should be interpreted as an approximate description.

### Experiment 2

#### Participants

Human subjects were recruited on New York University’s NY and Abu Dhabi (AD) campuses. A total of 24 right-handed, native English speakers participated in the experiment (eight males, 12 in AD). Four participants were excluded (one for not finishing the experiment because of a technical complication, one for excessive channel loss, and two for extreme noise during recording, rendering the data unusable). The age range of the remaining 20 participants was 19–42 years old (M = 26, SD = 6.46). All participants had normal or corrected to normal vision, no history of neurologic impairment and provided informed written consent. To mitigate our participant loss, we did not exclude participants based on behavioral accuracy. Participants were pseudo-randomly assigned one of three experimental lists, such that participants were equally divided over each experimental condition.

#### Stimuli

We developed a similar experimental paradigm as experiment 1, now manipulating the information value of the sentential context rather than manipulating properties of the modal items (modal base and force). We constructed 40 sets of bi-clausal English sentences, containing a causal relationship between the two parts. We contrasted the factual auxiliary verb *do* against the possibility modal verbs *may* and *might*, keeping modal force consistent across items.

**Figure 4.**
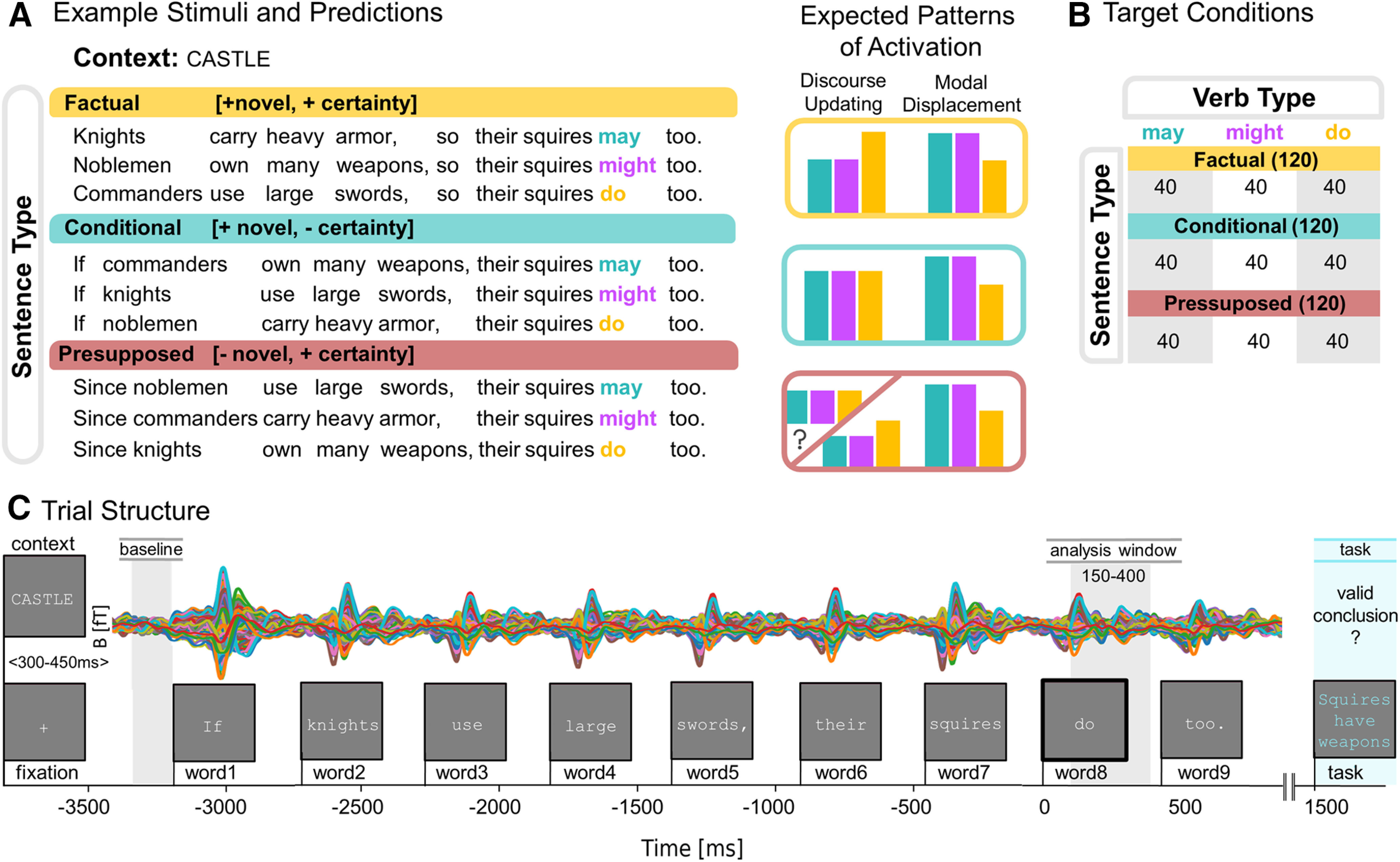
Experimental design and procedure experiment 2. ***A***, Example stimuli set and predictions. All stimuli were bi-clausal sentences of three different types: factual (*p* so *q*), conditional (if *p* → *q*), and presupposed (since *p* → *q*). These sentence types differed in whether they express information that is novel and certain (factual), novel and uncertain (conditional), or known and certain (presupposed). Each sentence contained either the factual verb *do* or the modal verbs *may* or *might*. Included are expected activation patterns for each verb per sentence type under processes of belief updating and modal displacement. We expect belief updating to take place in factual contexts but not in conditional contexts. For presupposed contexts, we had no clear predictions. Activity related to modal displacement is not expected to change across different sentential environments. ***B***, Experimental design with number of items per condition displayed between brackets (total = 360). The stimuli vary among two dimensions: Sentence Type (factual, conditional, and presupposed) and Verb (*may*, *might*, *do*). ***C***, Trial structure with evoked MEG responses in femtotesla (fT) from one participant. Procedure similar to experiment 1. Time windows for baseline correction (−3350 to −3200 ms) and statistical analysis (150–400 ms) are relative to the target verb (word8) onset.

Sentences differed in their informative content and came in three types: factual, e.g., “Knights carry large swords, so the squires do too,” which introduced novel information with certainty, conditional, e.g., “If knights carry large swords, the squires do too,” which introduced novel information with uncertainty (indicated by *if*), and presupposed, e.g., “Since knights carry large swords, the squires do too,” which introduced presumed to be known information (indicated by *since*) with certainty. The main manipulation (factual vs conditional) was added to test whether a possible effect of belief updating (expected to be present when encountering the factual target verb in the factual condition) disappeared if the information update built on uncertainty (conditional condition). For processing modal displacement, we did not expect a possible effect to be influenced by sentential certainty. We included the presupposed condition for exploratory purposes. Each sentence was preceded by a context word, indicating the theme of the upcoming sentence, e.g., “CASTLE,” to stay consistent with experiment 1, where utterances were preceded by a context sentence. Since experiment 2 did not vary modal base, we differentiated from experiment 1 by no longer embedding the target utterance into the perspective of a third person subject (used to bias toward modal base readings in experiment 1), to reduce sentence length. The complete stimulus design and predictions are displayed in Figure 4*A*. Each stimulus set consisted of nine sentences (3 × 3, Type: [factual, conditional, presupposed] × Verb: [*may*, *might, do*]) adding to a total of 360 sentences for all 40 stimuli sets (Fig. 4*B*).

All utterances were equal in length. Since we pursued a within-participants design and the different sentence conditions within a stimulus set differed minimally, we introduced controlled variance in the first clause of the utterance to make the paradigm seem less repetitive. We constructed three semantically related variants of the subject (e.g., *knights*, *noblemen*, and *commanders*) and main event (e.g., *carrying heavy armor*, *owning many weapons*, and *using large swords*) that were matched across conditions in a stimulus set so that each subject and action occurred in each of the nine conditions once. We made three different versions of the experiment such that across versions each condition occurred with all the subject and event variants. Sentential subjects denoted generic groups (e.g., *knights* or *loyal supporters*) and personal/company names (such as *Lisa* or *Facebook*).

#### Procedure

Before recording, we digitized the head shape of each participant with either a FastSCAN laser scanner or a FASTRAK 3D digitizer (Polhemus), following the same procedure as laid out in experiment 1. Before participants entered the MEG-room they received verbal instructions and did a short practice block of seven trials. Data collection took place in a magnetically shielded room using whole-head MEG system with 157 (NY) or 208 (AD) channels (Kanazawa Institute of Technology). Stimuli were projected onto a screen located above the participant. We made sure to keep the visual angle across both systems consistent, at ∼0.5° vertically.

In the experiment, participants were asked to silently read and comprehend causally linked sentences presented with PsychoPy (Peirce, 2009), font and background settings identical to experiment 1. First, a context word was displayed for 600 ms followed by a blank screen which display time varied between 300–450 ms. This jitter in display time was included to approximate the temporal variety in experiment 1 induced by self-paced reading of the context sentence. Then, a fixation cross (300 ms) followed and after a 300-ms blank screen the target sentence was presented using Rapid Serial Visual Presentation. Participants were presented with English sentences of nine words, one word at the time (300 ms on and 150 ms off). This was followed by a conclusion (displayed in blue) that was either a valid conclusion based on prior information (50%) or not. This task was designed such that participants had to pay close attention to the fine details of the target utterances. Forty percent of the questions specifically tapped into the certainty of the prior statement (e.g., the sentence “If knights own many weapons, their squires do too” followed by the valid conclusion “Potentially, the squires own many weapons” or invalid conclusion “The squires own many weapons”). Half of these certainty-based conclusions targeted the first clause of the sentence, while the other half targeted the second half. The other conclusions (60%) were more general e.g., “Knights have (no) squires.” The participant’s task was to press one button with their middle finger for conclusions that were valid and another button with their index finger if the conclusions were invalid, after which the next trial started. The participants were instructed to move and blink as little as possible during the task. The trial structure is displayed in Figure 4*C*.

The experiment consisted of 360 trials in total. The trials were divided into 9 separate blocks (containing one item per stimuli set) using a balanced Latin square design and randomized within blocks. Each block consisted of 40 sentences and was presented in two parts during the experiment, resulting in 18 blocks which took ∼3–5 min each. In between blocks, participants were informed about their overall accuracy. Participants were free to rest in between blocks and were paid $15 (NY) or 60 AED (AD) per hour.

#### Data acquisition

The same acquisition profile was maintained across both NY and AD systems, with settings as described for experiment 1. Preprocessing used the same software and pipeline as described for experiment 1. In total, 7% of the channels were interpolated because of being unresponsive or clearly malfunctioning (NY: 7–14 per participant; AD: 0–18 per participant). We extracted epochs from −3500 to 1200 ms relative to the onset of the target verb, which included the entire sentence, and rejected epochs containing signal amplitudes that exceeded a threshold of 3 pT (NY) or 2 pT (AD). The NY threshold is higher since that city and system has higher levels of overall ambient magnetic noise. In total, this resulted in a trial-rejection rate of 3.9% across all participants (NY: 5.0%; AD: 2.0%). Baseline correction was performed using data from −3350 to −3200 ms relative to the onset of the target verb, before the first word of the sentence. Source estimation followed the exact procedure as described for experiment 1. The inverse operator was calculated based on the covariance matrix from the 150-ms prestimulus baseline period of the cleaned trials.

#### Statistical analyses

##### Behavioral data

Overall accuracy per participant was based on responses to all 360 items. We also calculated the accuracy of the subset of task items (40%) probing the certainty of the target utterances.

##### MEG data

In order to compare our results from experiments 1 and 2, we conducted two analyses: an ROI analysis using the ROIs as defined for experiment 1 and a conceptual replication analysis searching for spatiotemporal clusters within a predefined region and time window based on the putative discourse updating effect of experiment 1.

##### ROI analysis

We used the same ROIs as used for the analysis of experiment 1, again assessing the effect of our experimental manipulations with a cluster-based permutation test (Maris and Oostenveld, 2007). We performed a temporal cluster-based permutation mass univariate 3 × 3 ANOVA with factors Sentence type and Verb. We based our analysis time window on the results of experiment 1, using a 150- to 400-ms time window after the target verb’s onset to replicate the effect found in the first experiment. Again, we equalized trial count across conditions. The number of trials per condition that were analyzed was on average 36 out of 40 for NY data (ranging from 31 to 38 per participant) and 38 out of 40 for the AD data (ranging from 34 to 40 per participant).

Our temporal permutation clustering test was performed with the same procedure as laid out for experiment 1 and corrected for comparisons across multiple ROIs (Benjamini and Hochberg, 1995).

##### Conceptual replication analysis

With the expectation of replicating the results from experiment 1, we limited our analysis to the factual sentence type condition. Then, we performed a spatiotemporal clustering analysis using the same procedure and settings as experiment 1. Informed by the results of experiment 1, instead of searching through the whole brain, the spatiotemporal analysis was now constrained to a predefined parcellation that combined regions in which we detected the effects of modal force in experiment 1. This ROI combined the right banks of STS and right superior parietal, supramarginal, superior temporal, inferior parietal and middle temporal gyri from the Freesurfer aparc parcellation. Like the ROI analysis, the time window of interest was 150–400 ms after the verb’s onset.

## Results

### Experiment 1

#### Behavioral results

The mean overall accuracy for the story congruency task was 83.1% (SD = 0.05), ranging from 71.6% to 92.5% across participants. The accuracy of the one third of the congruency task items that tapped into modality was 73.3% (SD = 0.08) ranging from 60.0% to 88.8% across participants, and was substantially lower than the accuracy of the other general items, which was 87.9% (SD = 0.05) ranging from 74.4% to 94.4% across participants.

#### ROI results

We ran a 2 (modal base: knowledge-based, rule-based) × 3 (modal force: possibility, necessity, factual) within-subjects temporal ANOVA for the ROIs specified for experiment 1. Since *may* and *must* differ in their lexical frequency across modal bases (*may* is high frequency as knowledge-based modal and low frequency as rule-based modal, *must* low frequency as knowledge-based modal and high frequency as rule-based modal, see Materials and Methods, Stimuli) we only report results that show consistent results across the modal force manipulation (knowledge-based and rule-based *may* or *must* patterning together) or the modal base manipulation (*may* and *must* patterning together).

**Figure 5.**
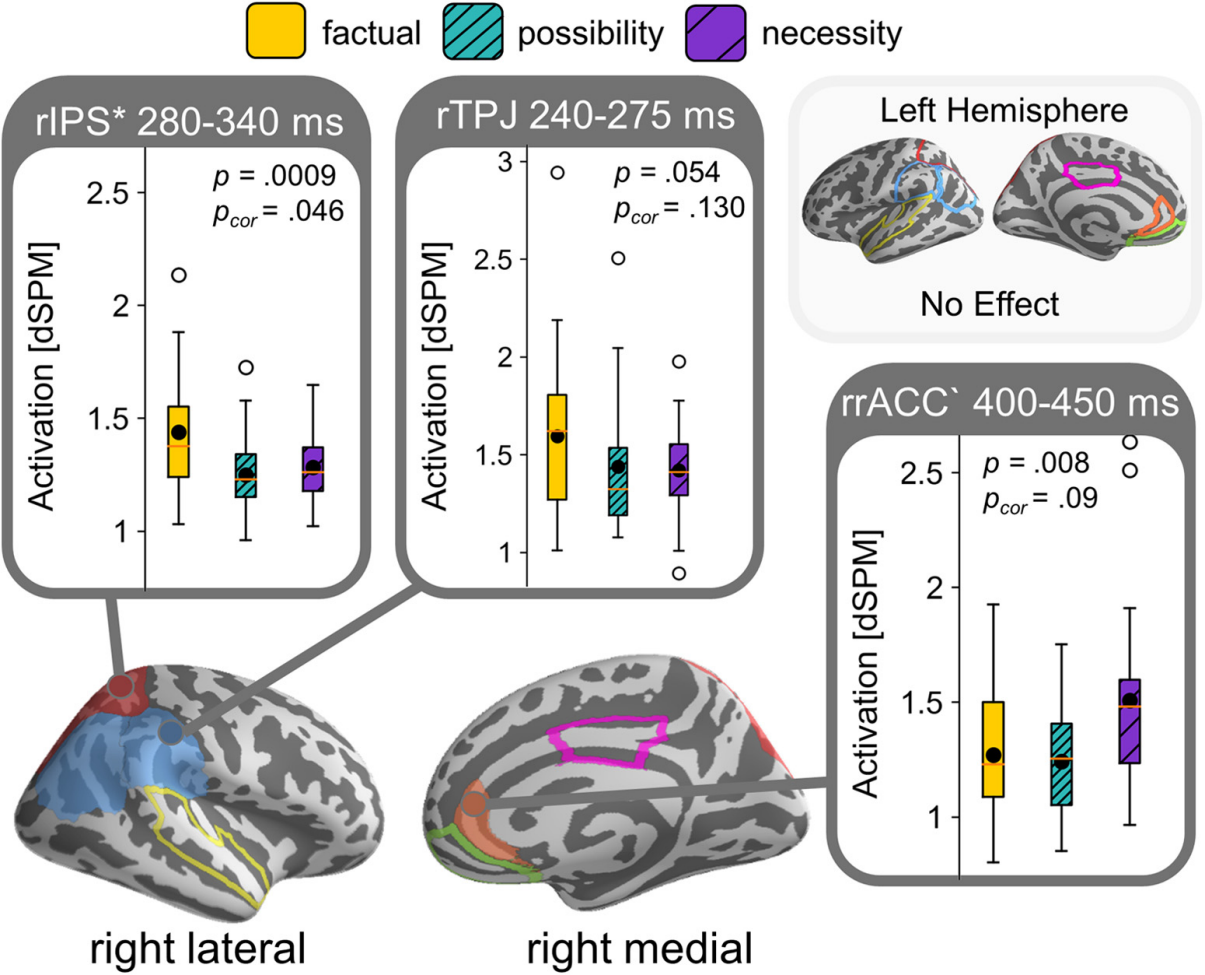
Summary ROI results experiment 1 showing a main effect for factual over modal (possiblity and necessity) conditions in rIPS and TPJ, and an increase in activation for necessity in the rrACC. Results are collapsed for modal base (knowledge-based and rule-based modals grouped together). Boxplots display estimated brain activity within the time window of the identified temporal clusters, black dots indicate mean activity. ROIs are outlined on brain and shaded when containing identified clusters. Clusters significant after correction comparison across multiple ROIs indicated with asterisk and with grave accent when trending.

**Figure 6.**
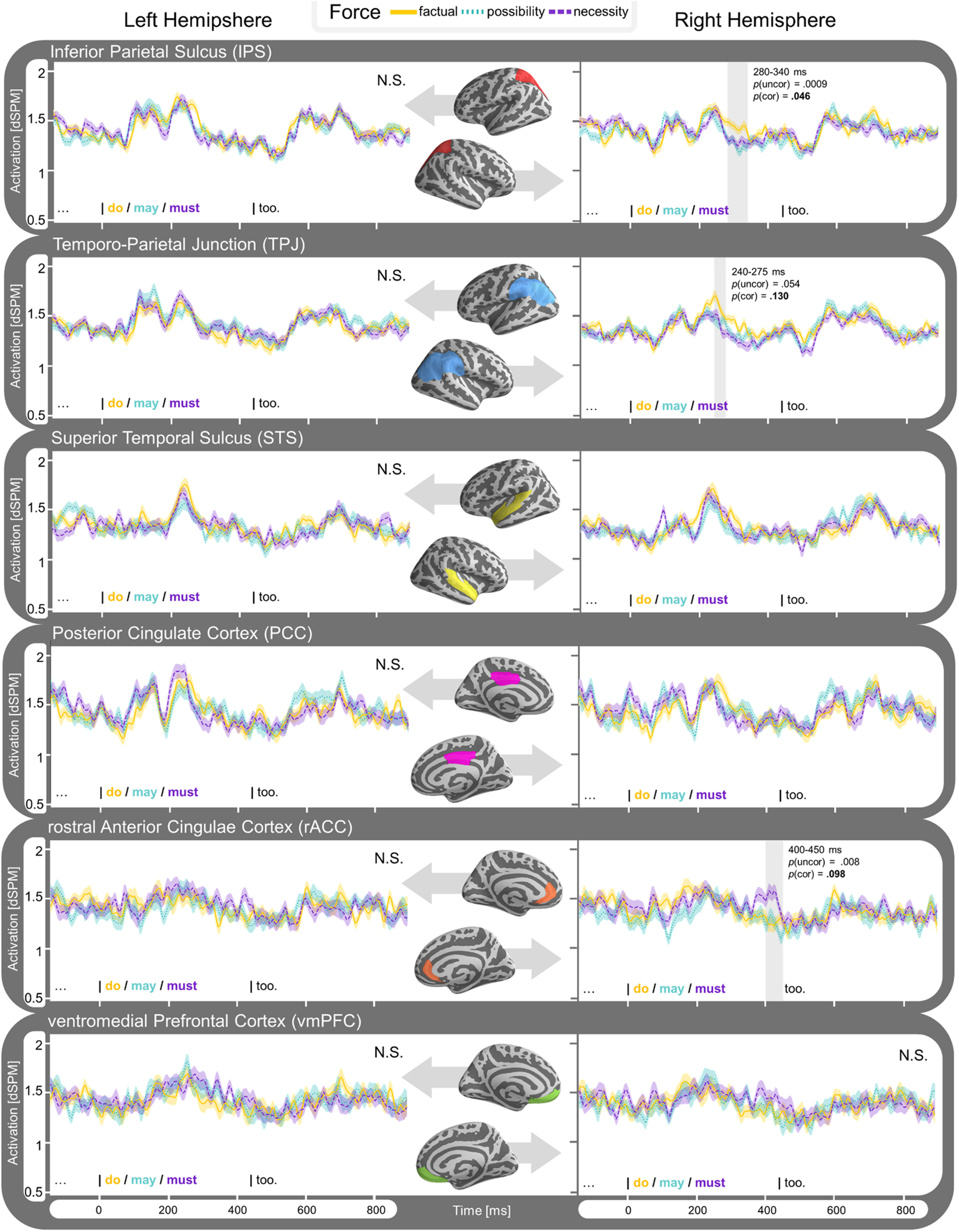
Time course of estimated average activity (dSPM) per modal force condition (factual, possiblity and necessity) for each ROI of experiment 1. Left hemisphere ROIs displayed on the left side, and right hemisphere on the right. Results collapsed for modal base (knowledge-based and rule-based modals grouped together). Detected clusters within time window 100–900 ms are highlighted and significance is indicated for the effect within the cluster (*p*_uncor_) and when corrected for comparison across multiple regions (*p*_cor_).

The ANOVA revealed a significant effect of modal force in the right intraparietal sulcus (rIPS) within our test window of 100–900 ms after the target verb’s onset (*p *=* *0.046), where the factual condition (*do*) elicited more activation than the modal (*may* and *must*) conditions. This temporal cluster extended from ∼280 to 340 ms. We observed a similar effect in a temporal cluster in the rTPJ around 240–275 ms, although this effect only survived multiple comparisons correction across time, not across multiple ROIs (uncorrected *p* = 0.054, *p* = 0.13). Additionally, we found a trending effect of modal force in the right rACC (rrACC), with increased activation for the necessity modal *must* over the other conditions (uncorrected *p *=* *0.008, *p *=* *0.099). We did not observe any other clusters in the remaining ROIs of the right hemisphere and did not observe any clusters in the left hemisphere. We summarized the ROI results in Figure 5 by depicting the activation patterns of the detected reliable clusters. The measured activity for each of the ROIs over our time window of interest are displayed in Figure 6.

#### Spatiotemporal results (whole brain)

**Figure 7.**
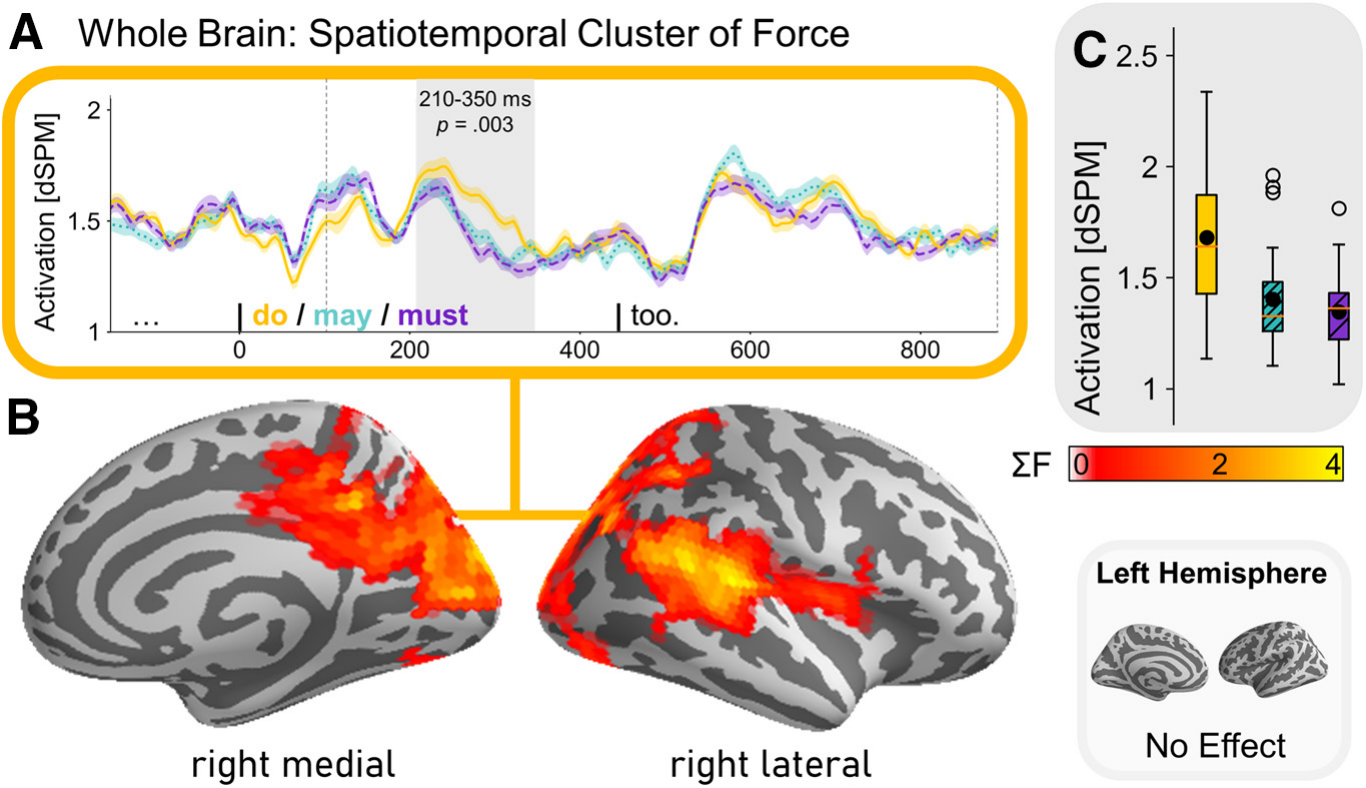
Identified spatiotemporal cluster of whole-brain analysis experiment 1. ***A***, Time course estimated brain activity (dSPM) split by modal force condition (factual, possiblity and necessity) and identified cluster in gray. Boundaries of analysis window (100–900 ms) are indicated by dashed lines. ***B***, FreeSurfer average brain shows spatial distribution of cluster, color shading indicating the sum of cluster-level *F* statistic (gained from cluster-based permutation test). ***C***, Boxplots display estimated brain activity (factual > modal) within the identified time window of the spatiotemporal cluster, black dots indicate mean activity.

A full-brain analysis revealed a significant effect for modal force, eliciting stronger activity for our factual condition over our modal conditions (*p* = 0.033) in our 100- to 900-ms time window. We detected a cluster between ∼210 and 350 ms centering around the rTPJ extending posteriorly over to the rIPS to the medial cortex, covering the cuneus, parts of the precuneus, and ending in the PCC (Fig. 7). The activation in this cluster reflects the activity we found for the effect of modal force in the rIPS and rTPJ of our ROI analysis. No other significant clusters were found.

### Experiment 2

#### Behavioral results

The mean overall accuracy for the conclusion validation task was 85.6% (SD = 0.09), ranging from 64.7% to 96.9% across participants. The accuracy of the subset of the validation task items that tapped into certainty was 83.7% (SD = 0.10) ranging from 57.6% to 95.2% across participants.

#### ROI results

**Figure 8.**
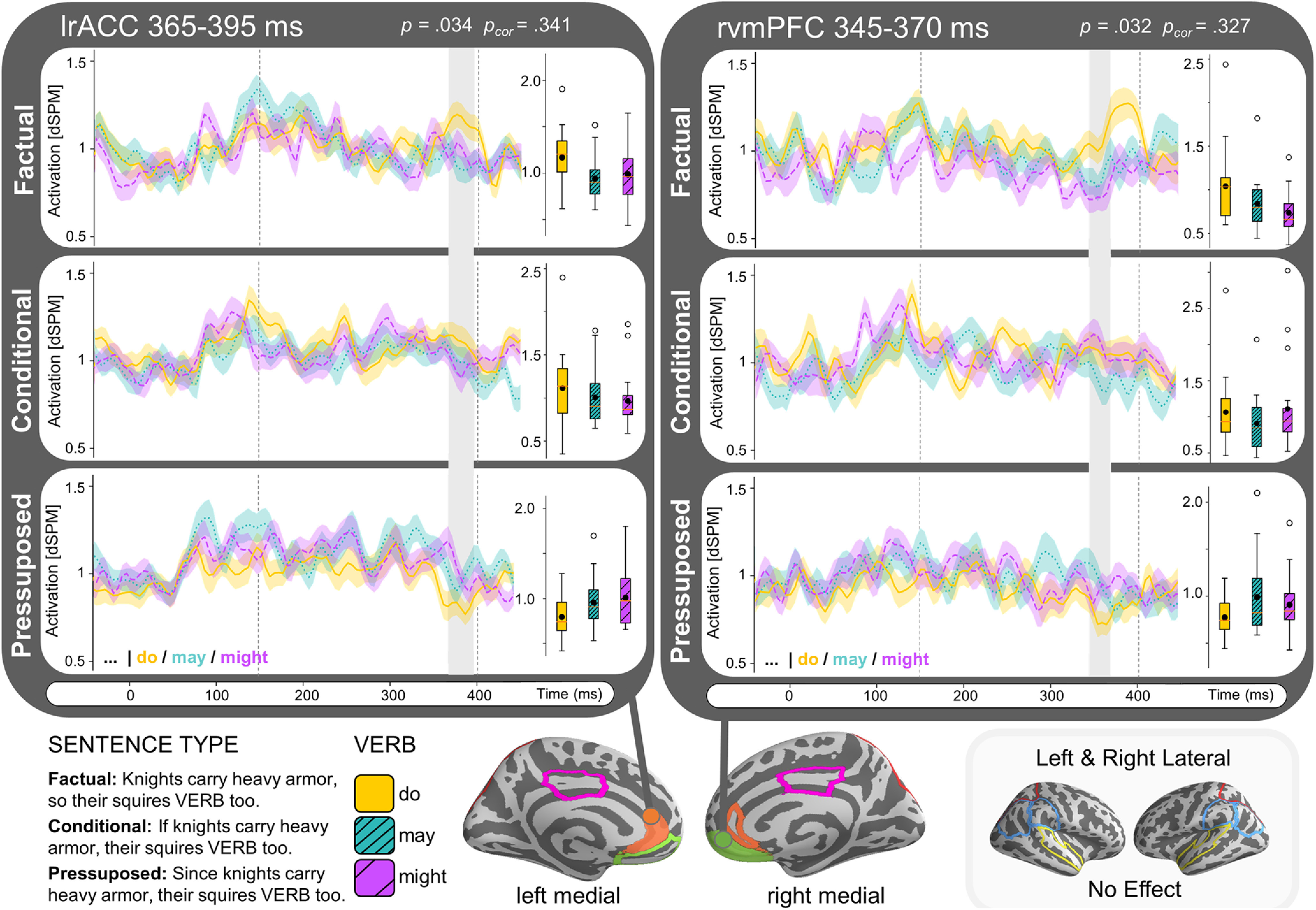
Time course estimated brain activity (dSPM) of reliable detected clusters from ROI analysis experiment 2. Both the lrACC and rvMPFC show an interaction between sentence type (factual, conditional, and presupposed) and verb (*do*, *may*, or *might*) with increased activation for *do* > *may/might* when embedded in factual sentences, and decreased activation for *do* < *may/might* in presupposed sentences. Boundaries of the analysis window (150–400 ms) are indicated by dashed lines, identified clusters displayed in gray. Boxplots display estimated brain activity within the time window of the identified temporal clusters, black dots indicate mean activity. ROIs are outlined on brain and shaded when containing identified clusters. Cluster effects are not significant after correction comparison across multiple ROIs. The effect in the lrACC was most prominent in the NY data while the effect in the rvMPFC was more prominent in the AD data (Extended Data Figure 8-1).

**Figure 9.**
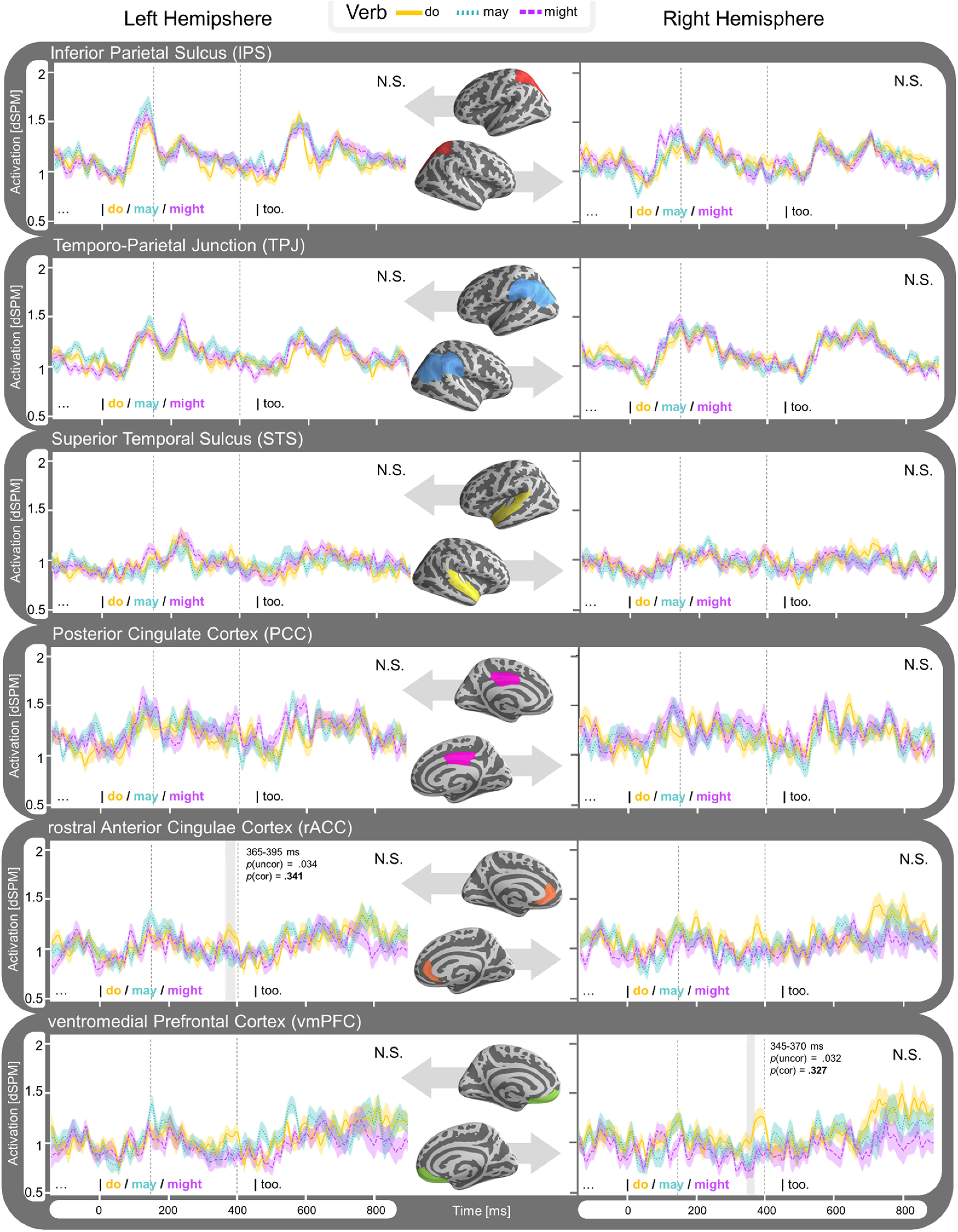
Time course of estimated average activity (dSPM) per ROI of experiment 2 for factual sentence type (*p* so *q*) split by verb condition (*do, might, must*). Left hemisphere ROIs displayed on the left side, and right hemisphere on the right. Detected clusters within time window 150–400 ms (indicated with dashed lines) are highlighted and significance is indicated for the effect within the cluster (*p*_uncor_) and when corrected for comparison across multiple regions (*p*_cor_).

We ran a 3 [Sentence Type: factual, conditional, presupposed] × 3 [verb: *may, might, do*] within-subjects temporal ANOVA for the same ROIs specified for experiment 1. We only observed effects that survived multiple comparisons correction across time, but not across multiple ROIs. The ANOVA revealed an interaction effect of verb and sentence type in the left rACC (lrACC) within our test window of 150–400 ms after the target verb’s onset (uncorrected *p* = 0.034, *p* = 0.341), where the factual condition (*do*) elicited more activation than the modal (*may* and *must*) conditions in factual sentences but not in conditional or presupposed sentences. In fact, in presupposed sentences the factual condition elicited less activity than the modal conditions. The temporal cluster reflecting this activity difference extended from ∼365 to 395 ms. We observed a similar effect in a temporal cluster in the right ventromedial PFC (rvMPFC) around 345–370 ms (uncorrected *p* = 0.032, *p* = 0.327). No other clusters were detected in any of the other ROIs. We summarized the ROI results in Figure 8 by depicting the time course of the detected reliable clusters. The effect in the lrACC was most prominent in the NY data, while the effect in the rvMPFC was more prominent in the AD data (Extended Data Fig. 8-1). The measured activity for each of the ROIs over our time window of interest in the factual sentential context (for comparison with Fig. 6) is displayed in Figure 9.

10.1523/ENEURO.0290-20.2020.f8-1 Extended Data Figure 8-1 Time course estimated brain activity (dSPM) of reliable detected clusters from ROI analysis experiment 2, displayed separately for the data collected in NY and the data collected in AD. Both the lrACC and rvMPFC show an interaction between sentence type (factual, conditional, and presupposed) and verb (*do*, *may*, or *might*) with increased activation for *do* > *may/might* when embedded in factual sentences, and decreased activation for *do* < *may/might* in presupposed sentences. The effect in the l**r**ACC was most prominent in the NY data, while the effect in the rvMPFC was more prominent in the AD data. Boundaries of the analysis window (150–400 ms) are indicated by dashed lines, identified clusters are displayed in grey. Boxplots display estimated brain activity within the time window of the identified temporal clusters, black dots indicate mean activity. ROIs are outlined on brain and shaded when containing identified clusters. Cluster effects are not significant after correction comparison across multiple ROIs. Download Figure 8-1, TIF file.

#### Conceptual replication results

We performed a spatiotemporal clustering test in the time window 150–400 ms in a ROI covering right lateral temporoparietal areas aiming to replicate the effect found in experiment 1. Unlike the results of experiment 1, a one-way ANOVA comparing activity within the verb condition (*do*, *may*, and *might*) in factual sentences detected no significant clusters in this area. This corroborates the results of the ROI analysis, in which we similarly found no difference in activity between the factual and modal verbs in the rIPS, TPJ, or STS.

## Discussion

In this work, we conducted two experiments to explore the neural correlates of modal displacement and discourse model updating during language comprehension. During natural discourse comprehension, the comprehender does not only integrate incoming factual information into an evolving discourse model but also entertains hypothetical situations denoted with modal utterances. We investigated how the brain distinguishes between factual and modal information.

Our stimuli contained short scenarios with two parts. The first part of the narrative established some property or habit that applied to one entity (e.g., “Knights carry heavy armor”), The second provided additional information about a second entity that was either factual (e.g., “the squires *do* too”) or modal (e.g., “the squires *may*/*must*/*might* too”). While the factual utterances indicated an actual change in situation, requiring the discourse representation to be updated, the modal utterances merely indicated a possible (uncertain) change. Our data showed that the factual condition elicited reliably stronger activation than the modal condition in right temporoparietal (experiment 1) and medial frontal regions (experiment 2). Below we discuss these increases as possible neural correlates of discourse model updating, elicited in the presence of updates that are certain (factual) but not for updates that are uncertain (modal).

### Neural correlates of discourse updating

Discourse updating, the operation of updating the mental representation of a situation, was modelled here as the attribution of a property to a new entity. Prior behavioral research has shown that mental representations of discourse are dynamically updated when presented with new facts (Glenberg et al., 1987; Morrow et al., 1989; Zwaan and Madden, 2004). Such model updating has been associated with increased activation in the mPFC, PCC, and temporoparietal areas (Fletcher et al., 1995; Ferstl et al., 2005; Xu et al., 2005; Speer et al., 2007; Yarkoni et al., 2008). In experiment 1, we found an increase in source-localized MEG responses for factual over modal statements. Specifically, activity increased in factual statements in the right lateral temporal and parietal hemisphere at ∼200–350 ms after target verb onset. This effect was most pronounced in the rIPS and less so in the rTPJ. This pattern of activity is compatible with behavioral findings on discourse updating. Factual utterances signal an actual change in the discourse, and when this information is incorporated into the comprehender’s mental representation this results in increased brain activity. In contrast, modal utterances only indicate a possible change of situation. Since the update is uncertain, situation model updating does not take place.

In experiment 2, we manipulated the broader sentential context in which novel factual and modal information was presented. In contrast to experiment 1, where the target sentence always built on a certain factual base, we now also presented the target utterance in conditionals that were hypothetical (uncertain, i.e., “If knights carry large swords…”) or presupposed (presumed to be common knowledge, i.e., “Since knights carry large swords…”). We expected discourse updating to only take place when the situational change is certain, and that embedding a factual update into a hypothetical conditional should prevent discourse updating from taking place because of the entire scenario being uncertain (Fig. 1).

While experiment 2 was designed to replicate the results from experiment 1 with our factual sentential context, we instead found that, this time, our ROI analysis (using the same ROIs as defined for experiment 1) revealed no differences in activity between factual and modal utterances in the right lateral hemisphere. This was confirmed by a replication analysis searching for spatiotemporal clusters targeting right lateral temporoparietal areas within the time window of 150–400 ms. Instead, we now found increased activity for factual over modal conditions in a temporal cluster in two adjacent areas: the lrACC and rvMPFC within our test window of 150–400 ms after the target verb’s onset. This effect only survived multiple comparisons correction across time, not across multiple ROIs. The hypothesis that this activation reflects discourse updating gains weight from the fact that we only observed this pattern of activity when the sentential context was factual (“Knights carry large swords, so their squires *do*/*may*/*might* too.”) but not when the sentential context was hypothetical (“If knights carry large swords, their squires *do*/*may*/*might* too.”). This would be in line with the idea that discourse model updating only takes place under certain situational changes, although such a conclusion has to be drawn with caution, as the results of experiment 2 were not that robust.

This presumed discourse updating effect resonates with prior behavioral studies on discourse updating and situation model maintenance. Discourse models representing a situation are dynamically updated as novel information indicating a change of situation comes along. As a consequence of model updating, “old” information that is no longer relevant to the here-and-now of a story is backgrounded, which is measurable in longer retrieval times in probe-recognition tasks compared with information that is still relevant to the current situation (Glenberg et al., 1987; Morrow et al., 1989; Zwaan and Madden, 2004). De Vega et al. (2007; de Vega and Urrutia, 2012) investigated whether this model updating also takes place when integrating hypothetical information, comparing accessibility after encountering factual (“As he had enough time, he went to the café to drink a beer”) and counterfactual utterances (“If he had enough time, he would have gone to the café to drink a beer”). De Vega et al. (2007) found evidence for discourse updating when integrating factual information but not for counterfactual information, leading them to conclude that the hypothetical meaning of counterfactuals does not contribute to the build-up of the discourse representation. This finding was corroborated in an event-related potential (ERP) study, where increased negativity after factual compared with counterfactual continuation utterances and reduced γ power following counterfactuals were taken to indicate that the counterfactual’s “as if” meaning is not integrated into the discourse (de Vega and Urrutia, 2012). Our results likewise suggest that mental model updating takes place for the integration of novel factual information, but not for hypothetical information as indicated by modality (*may/must/might*) or conditionality (*if…*).

This immediate sensitivity to the factual (*do*) versus hypothetical (*may/must*) contrast is in line with ERP findings showing rapid integration of contextual information in online processing. Prior context modulates the N400 component such that it takes more effort to retrieve lexical items compatible with the actual world in counterfactual utterances (where non-actual information is expected) than in factual or hypothetical utterances (Nieuwland and Martin, 2012; Kulakova and Nieuwland, 2016). Similarly, factive verbs like *know* presuppose complements compatible with the actual world, and when this expectation is violated it gives rise to P600 effects, taken to reflect conflict detection (Shetreet et al., 2019). While these ERP studies confirm that the brain is sensitive to the factual/hypothetical contrast during online processing, our results shed more light on when this information becomes available, possibly as soon as ∼200 ms after the target’s verb onset.

While the results of experiment 2 are less strong, they address some possible alternative explanations for the robust effect observed in experiment 1, which we hypothesized to reflect discourse updating. One might wonder whether a more low-level explanation could explain the observed activity increase for *do* over *may* and *must* in the first experiment, such as an inherent difference in lexical frequency (*do* is more frequent than *may* and *must*), polysemy (*may* and *must* are polysemous while *do* is not), or type of ellipsis (*do* ellipsis syntax may differ slightly from *may*/*must*). These alternative explanations are contradicted by the results of experiment 2, as we would have expected low-level effects like these to have been replicated in the same location and be insensitive to the experimental manipulation of our sentential context. Furthermore, we included the non-polysemous modal *might* to rule out the polysemy hypothesis. If the increase of factual over modal conditions in both experiments reflects discourse updating however, the question arises what caused the shift in location of this effect between experiments.

### Updating the representation of someone else’s mental state versus one’s own

In both of our experiments, we observed an increase for factual over modal expressions, henceforth “updating effect,” but the effect localized differently across the two experiments. In experiment 1, the updating effect was found in the rIPS and the adjacent rTPJ, while in experiment 2, we did not observe any effects in these specific areas. Instead, experiment 2 elicited a similar pattern of activity in medial frontal areas: the lrACC and rvMPFC. Both frontal medial and temporal parietal areas have been found to be involved in constructing and maintaining discourse representations in fMRI studies (Xu et al., 2005; Speer et al., 2007; Friese et al., 2008; Yarkoni et al., 2008; Ezzyat and Davachi, 2011). For example, Xu et al. (2005) investigated natural language comprehension at the level of words, sentences and narratives. When comparing visually presented isolated sentences and narratives, they observed robust response increases in several bilateral brain regions including the precuneus, medial prefrontal and dorsal temporo-parieto-occipital cortices. In a similar manipulation, contrasting unrelated sentences with coherent narratives, Yarkoni et al. (2008) found narrative-specific activation in the mPFC and additional neural contributions of posterior parietal regions supporting situation model construction and frontotemporal regions supporting situation model maintenance.

While both temporoparietal and frontal medial areas are part of the network engaged during narrative comprehension, one may wonder why experiment 2 did not replicate the discourse updating effect of experiment 1 in the same regions. The reason for this may be related to a change in materials between the experiments, altering whose mental representation is updated. In experiment 1, all target beliefs are attributed to a third person character, e.g., “But the king learns that the squires do too.” This third person character was included to enhance the contrast between the knowledge-based and rule-based modal readings, varying between authority and observer figures respectively. In contrast, experiment 2 lacked this third person character and embedding verb (“…, so the squires do too”) for the target manipulation to appear in conditional structures. By making this change in stimuli, we inadvertently changed whose mental state is updated during comprehension, someone else’s (experiment 1) or the participant’s own (experiment 2). When we represent someone else’s beliefs, we separate these from our own, as is evident from our ability to attribute false beliefs. For example, in the Introduction our example narrative contained the utterance “Pyramus quickly concludes she must have been devoured by the beast,” which allowed us to understand Pyramus thinks that his lover has died, although we know from the prior context that she is still alive. ToM encompasses the ability to represent someone else’s mental state separate from our own (Premack and Woodruff, 1978). ToM reasoning engages a network of brain regions, but it has been argued that particularly the rTPJ is involved in representing the mental state of others (Saxe and Kanwisher, 2003; Saxe and Wexler, 2005; Saxe and Powell, 2006; Vistoli et al., 2011) or reorienting attention (Decety and Lamm, 2007; Corbetta et al., 2008; Mitchell, 2008; Rothmayr et al., 2011). We tentatively suggest that the discourse updating effect in experiment 1 localized around the rTPJ because it involved updating a discourse representation separate from the comprehender’s own. Experiment 2 involved updating one’s own global representation and elicited activation in frontal medial regions. This is in line with studies finding medial prefrontal activity for tasks that require people to reflect on or introspect about their own mental states (Gusnard et al., 2001; Mitchell et al., 2005; Zhu et al., 2007). And this is also compatible with Ezzyat and Davachi (2011), who found that the bilateral vMPFC seemed especially engaged when integrating information within events, suggesting that this region could be sensitive to discourse updating.

Alternatively, it could be the case that the difference in results between experiment 1 and experiment 2 has to do with the different methods of contextualizing the target utterance. In experiment 1, the target sentence appeared after an initial context sentence that was read at the participant’s own pace. In experiment 2, the context before the target utterance merely consisted of one word introducing the general setting of the following utterance. While one may wonder whether these differences in context complexity (sentence vs word) and processing pace (self-paced vs timed) interfered with the baseline of the trial, it seems unlikely that this would be the cause for different results between experiments 1 and 2. Since all conditions within the experiments use the same baseline region, one would expect that any artifacts resulting from task effects are consistent across the different conditions of the experiments. Since we only compare conditions within experiments, the presence of an effect relative to other conditions cannot be because of a baseline effect (e.g., pressing a button). A more pressing question is whether the differences between the results of experiments 1 and 2 can be attributed to varying narrative complexity. In experiment 1, the (self-paced) context sentence established a property for one entity, and the target utterance then indicated that this property was also (possibly) shared by a second entity. In experiment 2, the target utterance consisted of two clauses, the first one establishing a (possible) property for one entity, while the second one stated that this property was (possibly) shared by a second entity. The entire target utterance was displayed with rapid serial visual presentation. Compared with experiment 1, experiment 2 thus allowed less time for participants to appreciate the initial situation (property being attributed to one entity) before updating this information (property also being attributed to second entity). An alternative explanation for our results could be that temporal parietal areas are more involved with constructing a larger discourse representation (coherence between sentences), while the medial frontal areas are more involved with initializing a discourse representation. This would be in line with Xu et al. (2005), who observed increased activity in the right hemisphere as contextual complexity increased.

An argument against this alternative hypothesis comes from recent work by Jacoby and Fedorenko (2020) investigating the neural correlates of expository discourse comprehension. While prior studies detected right temporal parietal engagement in comprehension of narratives (stories built around characters), expository texts (constituting facts about the real world) elicited no effect of discourse coherency in posterior ToM regions like the rTPJ (Jacoby and Fedorenko, 2020). This suggests that these regions only engage in coherence building for discourse in which you take someone else’s perspective. However, Jacoby and Fedorenko (2020) did find that the mPFC was sensitive to discourse coherency of expository texts. Since their expository texts were as complex as a narrative, it cannot be the case that the lack of engagement of the rTPJ observed for expository texts is because of a lack of discourse complexity. At the same time, the finding that the mPFC is sensitive to the coherence of expository texts suggests it could be involved in updating one’s own discourse beliefs.

### Neural correlates of modal displacement?

Before, we defined “modal displacement” as an operation that shifts our perspective from the immediate present to a hypothetical scenario. Several prior studies have investigated the neural correlates of utterances that involve hypothetical situations, but, as far as we know, no study has succeeded in isolating the neural mechanisms involved with the operation of modal displacement. Dwivedi et al. (2006) observed stronger responses for modal utterances (“it might end quite abruptly”) compared with factual utterances (“it ends quite abruptly”) and speculated this activity increase reflects the cost of mentally representing and comparing multiple possibilities. However, their study was not controlled for utterance length or complexity, leaving uncertain whether their observed activity increases were really because of the experimental manipulation. Another branch of neurolinguistic studies that investigates hypothetical meaning is research on the processing of counterfactuality, which engages parts of the default mode network such as the medial frontal and temporal lobes, the PCC, precuneus, and the lateral parietal and temporal lobes (Nieuwland, 2012; Urrutia et al., 2012; De Brigard et al., 2013; Kulakova et al., 2013; for recent overview, see Van Hoeck et al., 2015). Like modal constructions (e.g., “The monster might be big”), counterfactuals posit a hypothetical scenario (e.g., “If the monster were big…”). Unlike modal utterances, though, counterfactuals do not leave open any uncertainty about the actual state of affairs, rather they imply that the opposite is true (the monster is not big). On top of displacing from the here and now, the processing of counterfactual constructions involves keeping in mind two conflicting representations and inferencing the actual state of affairs. Any comparison between factual and counterfactual utterances (Urrutia et al., 2012) cannot separate these distinct processes.

Our study investigated modal displacement by minimally comparing factual and modal utterances. We found no reliable increases in neural activity when modal displacement occurred. However, the fact that we did find neural activation dissociating between the factual and modal condition suggests that participants processed the modal items as being different from the factual ones. Given that the increase in activation of factual over modal conditions takes place during the discourse integration of information indicating an actual change in situation, but not when integrating information regarding an uncertain (hypothetical) change, the most likely interpretation of our data is that this difference in activation reflects discourse updating.

However, if non-factual information does not get integrated into an existing situation model, the question remains how we *do* represent this information. The theoretical background for the current study was that modal displacement would involve the generation of multiple possibilities (Johnson-Laird, 1994; Iatridou, 2000; von Fintel, 2006; Kratzer, 2012). Intuitively, this would suggest that when presented with uncertainty, the comprehender postulates multiple mental representations of these different possibilities, the minimal one being a negated version (if squires *might* sit at round tables, this introduces the alternative possibility that maybe they do not). Considering multiple possibilities in parallel is thought to be cognitively demanding (Leahy and Carey, 2020), and we thus expected additional activity related to this operation. It is possible that this assumption was wrong, and that, for example, the decreased activity for modal utterances compared with factual utterances is indicative of modal displacement rather than discourse updating. However, it is difficult to gauge why this modal displacement is dependent on the sentential context and why we would find this correlate shifting in location across experiments. Alternatively, there might not be any correlates of representing multiple possibilities in the cortex at the level we investigated in this paper. Recently, Kay et al. (2020) found that possibility generation in rats involves a constant cycling between possible future scenarios in hippocampal neuron populations. At a constant cycling of 8 Hz, the cells alternated between encoding two different possible futures. The authors suggest this finding might extend to the representation of hypothetical possibilities in human brains, possibly extending to brain regions connected to the hippocampus.

Lastly, some have proposed that the representation of modality involves marking a representation with a symbolic operator, indicating that this representation can be neither ruled out nor added into the actual model (Leahy and Carey, 2020). This theory would not require people to actively postulate alternative situations, though the question remains how this uncertain information would be maintained and linked to the prior discourse if not incorporated into the existing situation model. For now, these questions are still open to future exploration.

### No effect of modal base and force

Our stimuli in experiment 1 were carefully designed to investigate the online comprehension of modal verbs varying in modal base (knowledge-based vs rule-based) and force (possibility vs necessity). However, we found no reliable effects of these manipulations. We did find an effect in the rrACC showing increased activation for necessity modals over the other conditions (Fig. 5), but this effect only survived multiple comparisons correction across time, not across multiple ROIs. The rostral ACC is, besides its involvement in ToM tasks, also argued to be involved in error processing and conflict resolution (Kiehl et al., 2000; Dreher and Grafman, 2003), suggesting that our effect may reflect some unnaturalness in our stimuli. The verb *must* requires strong evidence, but the surrounding context was made to be also compatible with weaker evidence (to allow for the appearance of *may*). Possibly, our stimuli contained too little evidence to naturally say *must*, eliciting increased activation in the rrACC when resolving this conflict.

In conclusion, this work investigated the integration of factual and modal information into short narratives. While the factual utterances indicated an actual change in situation, requiring the discourse representation to be updated, the modal utterances merely indicated a possible (uncertain) change as these utterances displaced from the narrative’s here-and-now. In a controlled within-subjects design, we measured source-localized MEG responses while participants integrated modal and factual information into a short narrative. While we did not find any regions of the brain more engaged by the modal conditions over the factual conditions (which could reflect engagement with modal displacement), we did find the opposite pattern of activation where certain brain regions elicited stronger activation for the factual over the modal condition. This increase in activation may be a neural correlate of mental discourse representation updating. This activity difference seems to go away as soon as the factual update is presented in an uncertain (conditional) sentential environment, supporting the idea that discourse updating only takes place when the change in the situation is certain. To our knowledge, this was the first attempt to explore the neural bases of modal processing. While we have established possible neural correlates of fact comprehension, the question of how uncertain information is integrated into a discourse representation remains open. We hope that our work establishes a starting point for further investigations of this phenomenon.

## Synthesis

Reviewing Editor: Christina Zelano, Northwestern University Decisions are customarily a result of the Reviewing Editor and the peer reviewers coming together and discussing their recommendations until a consensus is reached. When revisions are invited, a fact-based synthesis statement explaining their decision and outlining what is needed to prepare a revision will be listed below. The following reviewer(s) agreed to reveal their identity: Feng LIN.

This study used MEG and detailed behavioral tasks to examine the neural correlates of discourse processing. Both reviewers agreed that the study constitutes a significant advancement in the field, and that the experimental design and statistical analyses are technically sound. However, they raised several important points that should be addressed before further consideration. There are six items that need to be addressed:

1. left anterior Cingulate Cortex was not listed clearly in Table 1.

2. Although this study discussed a very interesting topic, the terminology is not easily understood by readers without proficient linguistic background. Some critical concepts, such as discourse updating, modal displacement, modal base and model force, were described by using instances rather than academic definitions. Please provide a list of concept definitions, either theoretical or operational, perhaps in the beginning of the Discussion.

3. The results of experiment 2 did not replicate the results of experiment 1. The authors explained the incompatibility through the influence of third person character. However, this factor was not introduced until the discussion. It would be better to describe the difference of person character in the Methods. Moreover, the major difference between the two experiments was not the materials but the context. Specifically, experiment 1 involved decision making at the beginning of a trial. This self paced decision could have interfered with the baseline of experiment 1. Another difference was that the context in experiment 1 required a sentence processing task (“Normally, only knights sit at the round table”), but the context in experiment 2 required a word reading task (“CASTLE”). The duration of the fixation cross (300ms) may not be long enough to avoid such task differences before the baseline of a trial. These two factors should also be discussed in paper.

4. The discussion should be expanded to include mention of other similar studies and address the speculative interpretations of the study relating to double representation of hypothetical scenarios. The authors suggest in several places that “there has been no systematic study of the neural bases of modal processing", “Since there is no prior neuroimaging work on modal displacement”. However, the authors overlooked research carried out by: Eugenia Kulakova y Mante Nieuwland; Mabel Urrutia, Manuel de Vega y Silvia Gennari; Ferguson HJ, Sanford AJ, Leuthold H.

5. Provide results on ERP Components because the authors only mention the time window

6. Clarify the differences between updating others’ beliefs vs updating one’s own beliefs, because attribution to a third person character alone is not clear
