# Supplementary material for: Neural Correlates of Modal Displacement and Discourse-Updating under (Un)Certainty
Source: eNeuro. 2021 Jan 12;8(1):ENEURO.0290-20.2020. doi: 10.1523/ENEURO.0290-20.2020 (PMC7810261; doi:10.1523/ENEURO.0290-20.2020)
Supplement: Extended Data Figure 3-1 — Details on controlled between-stimuli variation experiment 1. The target sentences were identical in structure, e.g., “But the king says that their squires may too” but varied in controlled manner in the following five ways. A, Overview of the variation in count of used connectives (and, but, and so) across modal bases. B, Variation of nouns (main subject) across modal base conditions in average length (in letters), average lexical frequency, average log lexical frequency, number of syllables, and number of morphemes. C, Variation of the determiners used to refer to the embedded subject: the, a long-distance pronoun (LD) referring to a referent in the prior context sentence or a short-distance pronoun (SD) referring to a referent in the target sentence. D, Variation of the elided VP across modal base conditions in average length (in words and letters), percentage of VPs that included verbs indicating a state (in contrast to an event), percentage of verbs taking two arguments (transitive) versus verbs that take one argument (intransitive), average syntactic node count [how many phrase nodes are present counting phrases containing a noun (NP), verb (VP), adjective (AP), preposition (PP), and infinitive (IP)] and average syntactic complexity (maximum amount of nodes opened at the same time), e.g., to see dusty books at the library includes five syntactic phrases [IP to [VP see [AP dusty [NP books]]]] [PP at the library] and has at most four nodes open at the same time. E1, List of different embedding verbs used with count of usage across modal bases. E2, Variation of embedding verbs used across modal base conditions in average length (in letters), average lexical frequency, average log lexical frequency, number of syllables, and number of morphemes. Download Figure 3-1, DOC file. [file enu-eN-NWR-0290-20-s02.doc]

**Figure 3-1**. Details on controlled between-stimuli variation Experiment 1.

A B E C D

e.g. Con | the | noun.sg | verb1 | that | det | Noun.pl | **Target** <elided VP> too

“But | the | king says that their squires | **may** <sit around the round table> too.”

**A**. Variation of connectives across bases **C.** Variation of determiners across bases

| Base | And | But | So | Total |
| --- | --- | --- | --- | --- |
| factual | 30 | 28 | 22 | 80 |
| rules | 30 | 28 | 22 | 80 |
| knowledge | 30 | 28 | 22 | 80 |
| Total | 90 | 84 | 66 | 240 |

^i^LD indicates long distance pronouns, pronouns that refer back to a referent in the context sentence, e.g. ‘Apparently knights overhear a lot of secrets in the castle. But the servant concludes that **their** squires must too’.

| Flavor | LD^i^ | SD^ii^ | the | Total |
| --- | --- | --- | --- | --- |
| *factual* | 22 | 22 | 36 | 80 |
| *rules* | 20 | 24 | 36 | 80 |
| *knowledge* | 24 | 20 | 36 | 80 |
| Total | 66 | 66 | 108 | 240 |

^ii^SD indicates a short distance pronoun, pronouns that refer back to a referent in the target sentence, e.g. ‘The bride's father is known to give boring speeches at receptions. But the bride hints that **her** brothers may too’.

**B**. Variation of nouns across modal base conditions

| Values | Rules | Knowledge |
| --- | --- | --- |
| Average Length (letters) | 6,8 | 7,6 |
| Average Freq_HAL | 83,9 | 87,20 |
| Average Log_Freq_HAL | 8984,9 | 8585,2 |
| Average #Syll | 2,25 | 2,5 |
| Average #Morph | 1,6 | 1,75 |

**D. Variation of Elided VP across modal base conditions (Av. = average)**

| Base | Av. Ellipsis Length (words) | Av. Ellipsis Length (letters) | Stative Verbs^i^ | Transitive Verbs^ii^ | Av. Node Count^iii^ | Av. Node Complexity^iv^ |
| --- | --- | --- | --- | --- | --- | --- |
| rules | 4,975 | 28,15 | 8% | 88% | 2,7 | 2,2 |
| knowledge | 4,775 | 27,9 | 15% | 88% | 2,925 | 2,55 |

^I^ Percentage of stative verbs (e.g. know, accept, live).

^ii^ Percentage of transitive verbs (with two arguments), 12 percent are intransitive verbs (taking one argument).

^iii^ Estimation of size syntactic structure by counting structural phrase nodes. Counted nodes are: noun phrase (NP), verb phrase (VP), predicate adjective phrase (AP), preposition phrase (PP), infinitival phrase (IP). NPs within APs or PPs were not counted. IPs were only counted in presence of the infinitival marker ‘to’. Adverbs, particles and attributive adjectives were ignored.

^iv^ Layers of embedded structure (complements). PPs are considered adjuncts not adding to complexity: e.g.

[IP [VP [NP]]] = counted as having 3 structural layers, [IP [VP] [PP]] = counted as having 2 structural layers.

**E1**. Variety and repetition of verb1 per condition.

Total 80 80 80

| Factual^i^ |  | Knowledge^ii^ |  | Rules^iii^ |  |
| --- | --- | --- | --- | --- | --- |
| acknowledges | 7 | assumes | 7 | agrees | 7 |
| discovers | 7 | believes | 7 | announces | 7 |
| hears | 7 | concludes | 7 | argues | 7 |
| knows | 7 | fears | 7 | assures | 6 |
| learns | 7 | figures | 6 | decides | 7 |
| notices | 7 | gathers | 5 | declares | 7 |
| observes | 7 | hints | 6 | determines | 7 |
| realizes | 6 | presumes | 7 | indicates | 7 |
| recognizes | 7 | reckons | 7 | informs | 7 |
| regrets | 5 | suspects | 7 | says | 7 |
| remembers | 6 | thinks | 7 | states | 7 |
| reveals | 7 | worries | 7 | warns | 4 |

^I^ Verbs of saying and directives/desideratives.

^ii^ Mental/emotion verbs.

^iii^ Perception/(semi)-factive verbs.

**E2**. Variation of verb1 per modal base condition.

| Values | Factual | Rules | Knowledge |
| --- | --- | --- | --- |
| Average Length (letters) | 7.75 | 6,75 | 7 |
| Average Freq_HAL | 7886.9 | 8624.2 | 8016.9 |
| Average Log_Freq_HAL | 141.6 | 111.53 | 111.598 |
| Average #Syll | 2.417 | 1.912 | 1.75 |
| Average #Morph | 2.25 | 2.33 | 2.25 |
