# Supplementary figures and images for: Neural Correlates of Modal Displacement and Discourse-Updating under (Un)Certainty
Source: eNeuro. 2021 Jan 12;8(1):ENEURO.0290-20.2020. doi: 10.1523/ENEURO.0290-20.2020 (PMC7810261; doi:10.1523/ENEURO.0290-20.2020)

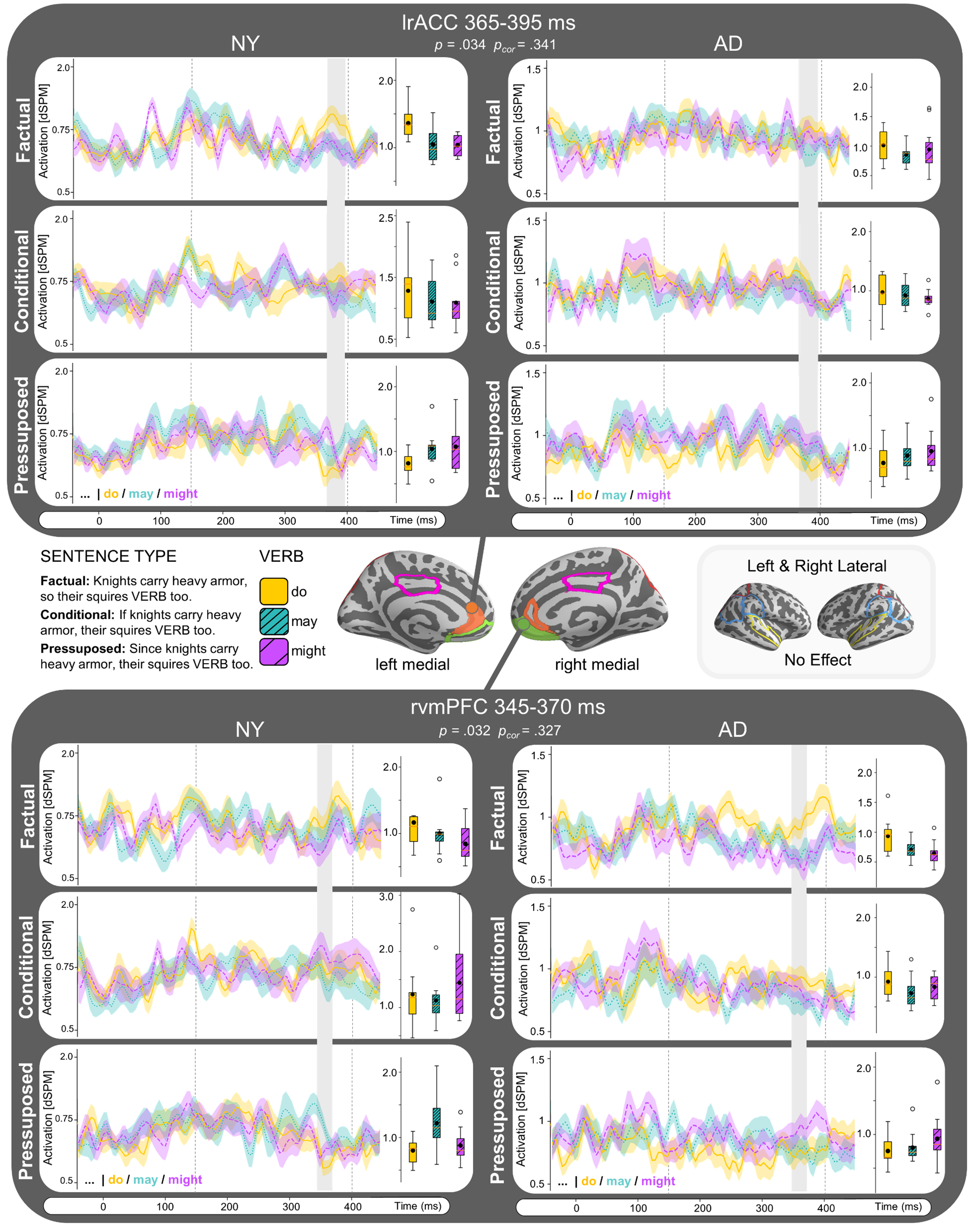

Supplement: Extended Data Figure 8-1 — Time course estimated brain activity (dSPM) of reliable detected clusters from ROI analysis experiment 2, displayed separately for the data collected in NY and the data collected in AD. Both the lrACC and rvMPFC show an interaction between sentence type (factual, conditional, and presupposed) and verb (do, may, or might) with increased activation for do > may/might when embedded in factual sentences, and decreased activation for do < may/might in presupposed sentences. The effect in the lrACC was most prominent in the NY data, while the effect in the rvMPFC was more prominent in the AD data. Boundaries of the analysis window (150–400 ms) are indicated by dashed lines, identified clusters are displayed in grey. Boxplots display estimated brain activity within the time window of the identified temporal clusters, black dots indicate mean activity. ROIs are outlined on brain and shaded when containing identified clusters. Cluster effects are not significant after correction comparison across multiple ROIs. Download Figure 8-1, TIF file. [file enu-eN-NWR-0290-20-s01.tif]
